# Effect of Thermal Gradient on Phase Gibbs Free Energy

**DOI:** 10.3390/ma19214676

**Published:** 2026-11-03

**Authors:** Ivaldo L. Ferreira

**Affiliations:** Faculty of Mechanical Engineering, Federal University of Pará (UFPA), Augusto Corrêa Avenue 1, Belém 66075-110, PA, Brazil; ileao@ufpa.br

**Keywords:** phase nucleation, thermodynamic equilibrium shift, phase nucleation formulation, Gibbs free energy, thermal gradient

## Abstract

Recent studies on phase nucleation have demonstrated a dependence on undercooling and thermal gradients during transient phase transformations. Assuming a Representative Elementary Volume (REV), in which thermodynamic functions and thermophysical properties are well-defined within the framework of continuum mechanics, an increase in the thermal gradient leads to higher surface energy and Gibbs free energy. This reflects the relationship between the bulk and the surface area of the nucleus. In this work, the surface stress (Σ_SL_), bulk free energy (ΔG_V_), surface free energy (ΔG_S_), configurational free energy (ΔG_C_), entropy (ΔS), and undercooling (ΔT) are calculated as functions of the thermal gradient (∇T) for the FCC_A1 phase of an Al-Cu-Mg-Fe alloy.

## 1. Introduction

Nucleation is a ubiquitous phenomenon that underpins a wide range of natural and industrial processes, including crystallization, melting, boiling, condensation, and the glass transition [1]. It can be examined from several theoretical perspectives, ranging from classical and statistical thermodynamics to molecular-dynamics and quantum-mechanical frameworks [2]. Classical nucleation theory (CNT) traces its origins to Fahrenheit’s observations of super-cooled water [3] and was subsequently placed on a firm thermodynamic foundation [4,5]. In its thermodynamic form, the theory expresses the reversible work of forming an embryo as a balance between a favorable bulk free energy change and the energetic cost of creating a new interface, from which a critical nucleus size and a Gibbs free energy barrier emerge; the steady-state nucleation rate then depends exponentially on this barrier through an Arrhenius-type law [6]. Although CNT remains the cornerstone for understanding crystal nucleation from the melt, it is not without limitations; in particular, it fails to describe vapor–liquid nucleation adequately [7,8].

A central prediction of the classical theory is that a liquid can be cooled substantially below its equilibrium melting temperature before crystallization sets in, with the undercooling providing the thermodynamic driving force for nucleation [9,10]. Turnbull’s droplet experiments on liquid metals established that homogeneous nucleation may require undercoolings of several hundred kelvin and, by inverting the measured nucleation frequencies, provided some of the earliest estimates of the crystal–melt interfacial energy [9]. In most practical situations, however, foreign surfaces, container walls, and impurities lower the barrier through heterogeneous nucleation, so that the observed undercooling is markedly smaller than the homogeneous limit [11,12,13]. Because the nucleation barrier scales with the cube of the interfacial energy relative to the square of the driving force, even modest uncertainties in the magnitude and anisotropy of this interfacial energy translate into large uncertainties in the predicted nucleation rate [14,15].

Non-classical approaches have therefore been developed. Density functional theory (DFT) treats nucleation as a problem of the intrinsic free energy functional of the density field and successfully models vapor–liquid as well as liquid–solid nucleation, even accounting for spinodal decomposition [16,17]. To overcome the sharp-interface assumption of the classical droplet model, diffuse-interface theory (DIT) incorporates the strong curvature dependence of surface tension, as well as the enthalpic and entropic contributions of the bulk phases within the interfacial region [16,17,18]. A further extension, diffuse-interface field theory (DIF), introduces multiple order parameters to distinguish between the initial and final phases, enabling predictions of nucleation in hydrocarbon liquids, liquid metals, and oxide glasses [19,20,21]. Complementary insight has come from atomistic simulation, where molecular dynamics studies now resolve nucleation pathways directly and increasingly reveal multistep and non-classical routes that the capillarity approximation cannot capture [22,23].

All of these theories rely on a set of auxiliary parameters to solve nucleation problems, but they do not provide a direct assessment of the underlying thermodynamic quantities. Consequently, despite its widespread use, CNT remains incomplete, and there is a clear need for a formulation that rests solely on thermodynamic variables. Such a framework would allow a rigorous description of the equilibrium dislocations that arise during phase nucleation under steep thermal gradients, without invoking any process-specific parameters.

The practical importance of these questions is most evident in alloy solidification, where nucleation is only the first step of a sequence that also encompasses interface kinetics, solute redistribution, and the morphological evolution of cellular and dendritic microstructures [24,25]. The selection of these microstructures is governed by the interplay between the thermodynamic driving force, the solid–liquid interfacial energy and its anisotropy, and the transport of heat and solute ahead of the moving front [24,26]. At high growth velocities, the interface departs from local equilibrium and solute trapping modifies the effective partition coefficient, an effect well described by continuous-growth kinetic models [27]. Quantitative prediction of these phenomena for multicomponent industrial alloys increasingly relies on coupling nucleation and growth descriptions to assess thermodynamic and atomic-mobility databases through the CALPHAD method [28].

In 2015, Kang shifted the thermodynamic equilibrium to calculate the surface energy for n-component compounds. In a boundary-phase model, the author treated the surface of a multicomponent solution as a distinct thermodynamic phase that equilibrates with the bulk while the total surface area is fixed. By enforcing equality of chemical potentials for each component (μ_i_ = μ_i_^(s)^), the surface tension σ emerges simply as the Gibbs energy difference between the surface and bulk phases divided by the molar surface area of the surface phase: σ = (g^(s)^ − g)/Ā^(s)^. This geometric relationship reproduces Butler’s early surface phase concept (1932) and is mathematically identical to the Constrained Gibbs Energy Minimization (CGEM) method of Pajarre et al. 2006 [29]. Because it requires only bulk and surface Gibbs energy descriptions—already available in CALPHAD databases—the relation can be implemented directly in CALPHAD codes to compute σ for any composition. The formulation highlights how pure-component surface tensions (σ_i_°), molar surface areas (A_i_°), bulk excess Gibbs energy, and surface excess Gibbs energy together dictate both the macroscopic surface tension and the equilibrium surface composition, providing a clear, unified framework for predicting surface properties of complex solutions. This procedure was successfully used to calculate thermodynamic properties, but it resulted in complex geometrical relationships [30]. A procedure for shifting the thermodynamic equilibrium based on the nucleation formulation is necessary for the calculation of thermodynamic functions and thermophysical properties.

Noteworthy contributions have been made by Koukkari and Pajarre [29]. However, both studies neglect surface physical terms such as the surface free energy potential and the configurational free energy. Their optimizations rely on an energy potential that is prescribed externally to the nucleation model, whereas contemporary approaches formulate the problem directly in terms of potentials.

Recently, the thermal field tensor was defined in terms of an equilibrium shift to assess its primitive thermodynamic variables by applying a thermal field gradient, based on the combination of the first and second laws of thermodynamics. Theoretical simulations [31] for the Gibbs–Thomson coefficient were used to predict the experimental microstructure evolution, which experimentally confirmed the theoretical observation that nucleation phenomena are second order in the nucleation radius [32,33]. Nevertheless, a unified description that expresses the nucleation barrier and the associated thermodynamic functions solely in terms of measurable thermodynamic variables, and that explicitly resolves how an imposed thermal gradient reshapes the bulk, surface, and configurational free energy contributions, remains lacking [22].

Recent advances in computational materials science have increasingly focused on developing physically interpretable and computationally efficient frameworks capable of accurately predicting complex material behavior. Machine learning has emerged as a powerful tool for accelerating the design and optimization of advanced alloys by establishing quantitative relationships between composition, microstructure, processing parameters, and mechanical properties while preserving physical interpretability. In parallel, significant efforts have been devoted to constructing generalized constitutive formulations that accurately describe anisotropic plastic deformation through convex, data-driven yield criteria applicable to a wide range of materials without requiring material-specific functional forms. These developments demonstrate a clear trend toward integrating physics-based principles, data-driven methodologies, and advanced numerical algorithms into unified computational frameworks, thereby improving predictive capability, computational robustness, and transferability across multiple classes of engineering materials and loading conditions [34,35].

In this study, the thermal-field tensor and the nucleation radius [31] are expressed in terms of the bulk, surface, and configurational free energies, in order to analyze how thermal gradients affect the free energy contributions. General formulations for the surface free energy, the surface-potential free energy, the surface entropy, and the surface-potential entropy are also derived.

## 2. Problem Formulation

The main objective of this paper is to rewrite the nucleation formulation equations, originally expressed in terms of primitive thermodynamic variables [31], in terms of the bulk free energy $\Delta G_{V}$, surface free energy $\Delta G_{S}$, and configurational free energy $\Delta G_{C}$, with the aim of analyzing how these energies are affected by the thermal field gradient ΔT.

### 2.1. A Proper Field for Phase Transformation

The first law of thermodynamics for a system can be written as

(1) δQ−δW=dE,

which means that the heat exchange between hot and cold reservoirs can be posed mathematically as $Q=f\left(E,W\right)$. Energy E is a point function and, heat Q and work W are path-dependent quantities, i.e., line functions.

Let us then define a new field, using tensor calculus, dE≡∂E,i, in relation to the temperature T primitive variable, we can express the first law of thermodynamics as

(2) $$\nabla \left[\frac{\partial T}{\delta Q}\left(Q-W\right)\right]=\nabla \left[\frac{\partial T}{\delta Q}E\right],$$

This new field, called the thermal field gradient $\nabla T_{pi}$, is a path-independent field given by the following equation:

(3) $$\nabla T_{pi}=\nabla \left[\frac{\partial T}{\delta Q}\left(Q-W\right)\right]=\nabla \left[\frac{\partial T}{\delta Q}E\right],$$

A path-dependent thermal field gradient $\nabla T_{pi}$ and transformation relationship can be obtained by rearranging Equation (3) in terms of external work as

(4) $$\nabla T_{pd}=\nabla \left[\frac{\partial T}{\delta Q}Q\right]=\nabla \left[\frac{\partial T}{\delta Q}E\right]+\nabla \left[\frac{\partial T}{\delta Q}W\right],$$

and

(5) $$\nabla T_{pd}=\nabla \left[\frac{\partial T}{\delta Q}Q\right]=\nabla T_{pi}+\nabla \left[\frac{\partial T}{\delta Q}W\right],$$

Analyzing the first law of thermodynamics for heat and work over a given path $C_{1}=TS$, as shown in Figure 1 in general coordinates X^i^, and applying Green’s theorem, we obtain

(6) $$Q-W={\left.Q\right|}_{Pi}+{\left.\oint_{TS}\left(\frac{{\partial Q}_{1}}{\partial x}dx^{1}+\frac{\partial Q_{2}}{\partial x}dx^{2}\right)\right|}_{Pd}-\left[{\left.W\right|}_{Pi}+{\left.\oint_{TS}\left(\frac{{\partial W}_{1}}{\partial x}dx^{1}+\frac{\partial W_{2}}{\partial x}dx^{2}\right)\right|}_{Pd}\right]=E,$$

hence,

(7) $$\oint_{TS}\left(\frac{{\partial Q}_{1}}{\partial x}dx^{1}+\frac{\partial Q_{2}}{\partial x}dx^{2}\right)-\oint_{TS}\left(\frac{{\partial W}_{1}}{\partial x}dx^{1}+\frac{\partial W_{2}}{\partial x}dx^{2}\right)=0,$$

Performing a permutation of the tensor calculus indices on the LHS and RHS of Equation (4), we obtain the following relationship:

(8) $$\nabla \left(\frac{\partial T}{\delta Q}Q\right)=\nabla \left[\frac{\partial T}{\delta Q}E\right]+\nabla \left[\frac{\partial T}{\delta Q}W\right]≅\nabla \left[\frac{\partial T}{\partial E+\delta W}E\right]+\nabla \left[\frac{\partial T}{\partial E+\delta W}W\right],$$

By the first law, the relation $\delta Q=f\left(dE,\delta W\right)$, treats E and W as independent variables, so that $\nabla \left[\frac{\partial T}{\delta W}E\right]$ and $\nabla \left[\frac{\partial T}{\partial E}W\right]$ may be neglected, as experimentally observed [32,33]. Finally, the path-dependent thermal field gradient ∇T (measured in K m^−1^) can be defined as

(9) $$\nabla T_{pd}=\nabla \left(\frac{\partial T}{\delta Q}Q\right)=\nabla \left[\frac{\partial T}{\delta Q}E\right]+\nabla \left[\frac{\partial T}{\delta Q}W\right]≅\nabla \left[\frac{\partial T}{\partial E}E\right]+\nabla \left[\frac{\partial T}{\delta W}W\right],$$

as recently derived [31].

The thermal field gradient ∇T can be written in terms of energy components, i.e., $E=U+K+E_{P}+\Sigma_{other}$,

(10) $$\nabla T≅\nabla \left[\frac{\partial T}{\partial E}E\right]+\nabla \left[\frac{\partial T}{\delta W}W\right]≅\nabla \left[\frac{\partial T}{\partial E}\left(U+K+E_{P}+\Sigma_{other}\right)\right]+\nabla \left[\frac{\partial T}{\delta W}W\right]$$

and the indices permutation can also be applied,

(11) $$\nabla T≅\nabla \left[\frac{\partial T}{\partial E}E\right]+\nabla \left[\frac{\partial T}{\delta W}W\right]≅\nabla \left[\frac{\partial T}{\left(\partial U+\partial K+\partial E_{P}+{\partial \Sigma }_{other}\right)}\left(U+K+E_{P}+\Sigma_{other}\right)\right]+\nabla \left[\frac{\partial T}{\delta W}W\right],$$

The thermal field gradient ∇T can be written in terms of energy components as

(12) $$\nabla T≅\nabla \left[\frac{\partial T}{\partial E}E\right]+\nabla \left[\frac{\partial T}{\delta W}W\right]≅\nabla \left[\frac{\partial T}{\partial U}U\right]+\nabla \left[\frac{\partial T}{\partial K}K\right]+\nabla \left[\frac{\partial T}{\partial E_{P}}E_{P}\right]+\nabla \left[\frac{\partial T}{{\partial \Sigma }_{other}}\Sigma_{other}\right]+\nabla \left[\frac{\partial T}{\delta W}W\right],$$

and as a function of internal energy U components, e.g., $U_{transl}+U_{vib}+U_{rot}+U_{elect}+U_{mag}+U_{other}$,

(13) $$\nabla T≅\nabla \left[\frac{\partial T}{\partial U}\left(U_{transl}+U_{vib}+U_{rot}+U_{elect}+U_{mag}+U_{other}\right)\right]+\nabla \left[\frac{\partial T}{\partial K}K\right]+\nabla \left[\frac{\partial T}{\partial E_{P}}E_{P}\right]+\nabla \left[\frac{\partial T}{{\partial \Sigma }_{other}}\Sigma_{other}\right]+\nabla \left[\frac{\partial T}{\delta W}W\right],$$

An important classical form of the thermal field for phase transformation is provided by the Gibbs–Duhem equation, a particular case of the thermal field gradient ∇T, which is based on the first law of thermodynamics [31,32,33]; the slope is expressed in terms of the molar specific heat capacity, accounting for all contributions to the internal energy $\frac{\partial T}{\partial U}=\frac{1}{C_{m}}$,

(14a) $$\nabla T≅\nabla \left[\frac{\partial T}{\partial U}\left(A\gamma \right)\right]=\nabla \left\{\frac{\partial T}{\partial U}\left[TS+N^{Surf}\mu -A_{0}\left(\sum_{\alpha \beta }\left(\Sigma_{\alpha \beta }-\gamma \delta_{\alpha \beta }\right)\varepsilon_{\alpha \beta }\right)+U_{mag}+U_{others}\right]\right\}+\nabla \left[\frac{\partial T}{\partial K}K\right]+\nabla \left[\frac{\partial T}{\partial E_{P}}E_{P}\right]+\nabla \left[\frac{\partial T}{{\partial \Sigma }_{other}}\Sigma_{other}\right]+\nabla \left[\frac{\partial T}{\delta W}W\right],$$

and finally,

(14b) $$\nabla T≅\nabla \left[\frac{\partial T}{\partial U}\left(A\gamma \right)\right]=\nabla \left\{\frac{1}{C_{m}}\left[TS+N^{Surf}\mu -A_{0}\left(\sum_{\alpha \beta }\left(\Sigma_{\alpha \beta }-\gamma \delta_{\alpha \beta }\right)\varepsilon_{\alpha \beta }\right)+U_{mag}+U_{others}\right]\right\}+\nabla \left[\frac{\partial T}{\partial K}K\right]+\nabla \left[\frac{\partial T}{\partial E_{P}}E_{P}\right]+\nabla \left[\frac{\partial T}{{\partial \Sigma }_{other}}\Sigma_{other}\right]+\nabla \left[\frac{\partial T}{\delta W}W\right],$$

### 2.2. A Novel Formulation for Phase Nucleation

The critical total free energy Δ*G* in Ferreira [31] can be deduced in terms of the bulk free energy $\Delta G_{V}$ and surface free energy $\Delta G_{S}$ and the nucleation radius r as follows:

(15) $$\Delta G=\left(\frac{1}{3}\pi r_{C}^{3}\Delta S_{V}\Delta T+\pi r_{C}^{2}\gamma_{SL}\right)f\left(\theta \right)=\left(\frac{1}{3}\pi r_{C}^{3}\Delta G_{V}+\pi r_{C}^{2}{\Delta G}_{S}\right)f\left(\theta \right),$$

where $f(\theta )=2-3cos\theta +{cos}^{3}\theta$. The solid–liquid interfacial energy, for the sake of generality, is expressed in terms of the surface free energy $\gamma_{SL}\equiv \Delta G_{S}$. By differentiating the critical free energy with respect to the critical radius $r_{C}$ and setting the derivative to zero, one obtains a quadratic (parabolic) equation for the extremal value of $r_{C}$.

(16) $$\left[\left(\frac{\partial \Delta S_{V}}{\partial r}\Delta T+\Delta S_{V}\frac{\partial \Delta T}{\partial r}\right)f\left(\theta \right)+\Delta S_{V}\Delta T\frac{\partial f\left(\theta \right)}{\partial r}\right]r_{C}^{2}+3\left[\left(\Delta S_{V}\Delta T+\frac{{\partial \gamma }_{SL}}{\partial r}\right)f\left(\theta \right)+\gamma_{SL}\frac{\partial f\left(\theta \right)}{\partial r}\right]r_{C}+6\gamma_{SL}f\left(\theta \right)=0,$$

A second-order critical radius $r_{C}^{2nd}$ can be derived from the nucleation theory written as a function of primitive thermodynamic variables in terms of the bulk, surface, and configurational free energy $\Delta G_{V}$, $\Delta G_{S}$, and $\Delta G_{C}$, respectively, as given,

(17) $$r_{C}^{2nd}=-\frac{3}{2}\frac{\left[\left(\Delta S_{V}\Delta T+\frac{{\partial \gamma }_{SL}}{\partial r}\right)+\frac{\gamma_{SL}}{f\left(\theta \right)}\frac{\partial f\left(\theta \right)}{\partial r}\right]}{\left[\left(\frac{\partial \Delta S_{V}}{\partial r}\Delta T+\Delta S_{V}\frac{\partial \Delta T}{\partial r}\right)+\frac{\Delta S_{V}\Delta T}{f\left(\theta \right)}\frac{\partial f\left(\theta \right)}{\partial r}\right]}=-\frac{3}{2}\frac{\left[\left(\Delta S_{V}\Delta T+\frac{{\partial \gamma }_{SL}}{\partial r}\right)f\left(\theta \right)+\gamma_{SL}\frac{\partial f\left(\theta \right)}{\partial r}\right]}{\left[\frac{\partial \left(\Delta S_{V}\Delta Tf\left(\theta \right)\right)}{\partial r}\right]}=-\frac{3}{2}\frac{\left[\Delta G_{V}+\frac{\partial \Delta G_{S}}{\partial r}+\Delta G_{C}\right]}{\frac{\partial \Delta G_{V}}{\partial r}},$$

and the first-order approximation of the critical radius $r_{C}^{\left(1st\right)}$ can be expressed as follows:

(18) $$r_{C}^{1st}=-\frac{2\gamma_{SL}f\left(\theta \right)}{\left[\left(\Delta S_{V}\Delta T+\frac{{\partial \gamma }_{SL}}{\partial r}\right)f\left(\theta \right)+\gamma_{SL}\frac{\partial f\left(\theta \right)}{\partial r}\right]}=-\frac{2\Delta G_{S}}{\Delta G_{V}+\frac{\partial \Delta G_{S}}{\partial r}+\Delta G_{C}},$$

which is similar to that expected from Classical Nucleation Theory (CNT), where $r_{C}^{CNT}=-\frac{2\Delta G_{S}}{\Delta G_{V}}$.

A second-order thermal-field tensor $\Gamma^{\left(2nd\right)}$ can be obtained by maximizing the total Gibbs free energy as follows:

(19) $$\Gamma^{2nd}=-\frac{3}{4}\frac{\left[\left(\Delta S_{V}\Delta T+\frac{{\partial \gamma }_{SL}}{\partial r}\right)+\frac{\gamma_{SL}}{f\left(\theta \right)}\frac{\partial f\left(\theta \right)}{\partial r}\right]\Delta T}{\left[\left(\frac{\partial \Delta S_{V}}{\partial r}\Delta T+\Delta S_{V}\frac{\partial \Delta T}{\partial r}\right)+\frac{\Delta S_{V}\Delta T}{f\left(\theta \right)}\frac{\partial f\left(\theta \right)}{\partial r}\right]}=-\frac{3}{4}\frac{\left[\left(\Delta S_{V}\Delta T+\frac{{\partial \gamma }_{SL}}{\partial r}\right)f\left(\theta \right)+\gamma_{SL}\frac{\partial f\left(\theta \right)}{\partial r}\right]}{\frac{1}{\Delta T}\left[\frac{\partial \left(\Delta S_{V}\Delta Tf\left(\theta \right)\right)}{\partial r}\right]}=-\frac{3}{4}\frac{\left[\Delta G_{V}+\frac{\partial \Delta G_{S}}{\partial r}+\Delta G_{C}\right]}{\frac{\partial \Delta S_{V}}{\partial r}},$$

The first-order approximation of the thermal-field tensor $\Gamma^{\left(1st\right)}$ is given by

(20) $$\Gamma^{1st}=-\frac{\gamma_{SL}f\left(\theta \right)}{\left[\left(\Delta S_{V}\Delta T+\frac{{\partial \gamma }_{SL}}{\partial r}\right)f\left(\theta \right)+\gamma_{SL}\frac{\partial f\left(\theta \right)}{\partial r}\right]}\Delta T=-\frac{\Delta G_{S}}{\Delta G_{V}+\frac{{\partial \Delta G}_{S}}{\partial r}+\Delta G_{C}}\Delta T=-\frac{\Delta G_{S}}{\frac{1}{\Delta T}\left[\Delta G_{V}+\frac{\partial \Delta G_{S}}{\partial r}+\Delta G_{C}\right]}=-\frac{\Delta G_{S}}{\Delta S_{V}+\frac{{\partial \Delta S}_{S}}{\partial r}+\Delta S_{C}},$$

The nucleation rate I derived by Ferreira (2024) [31] is given by

(21) $$I=\frac{DA}{\lambda^{4}}\frac{N}{V}\exp\left(\frac{\Delta G_{C}}{\Delta G_{C,Eq}}\right),$$

where D is the self-diffusion coefficient in $m^{2}s^{-1}$, N/V is the density of modes in the new phase in $modes\cdot m^{-3}$, λ is the distance an atom must jump from a phase to the nucleating new surface area in m, A is the surface area of the new phase in $m^{2}$.

### 2.3. A General Phase Transformation Equation

Ferreira’s newly derived thermal-field tensor **Γ**, as given by Equation (19), which embodies the second law of thermodynamics, and the thermal field T, according to Equation (4), representing the first law, can be combined into a compact expression for predicting phase nucleation [31,32,33]:

(22) $$\Gamma =-\frac{3}{4}\frac{\left[\Delta G_{V}+\frac{\partial \Delta G_{S}}{\partial r}+\Delta G_{C}\right]}{\frac{\partial \Delta S_{V}}{\partial r}}=A\cdot \nabla T=A\cdot \left\{\nabla \left[\frac{\partial T}{\delta Q}E\right]+\nabla \left[\frac{\partial T}{\delta Q}W\right]\right\}\;=A\cdot \left\{\nabla \left[\frac{\partial T}{\partial U}\left(U_{transl}+U_{vib}+U_{rot}+U_{elect}+U_{mag}+U_{other}\right)\right]+\nabla \left[\frac{\partial T}{\partial K}K\right]+\nabla \left[\frac{\partial T}{\partial E_{P}}E_{P}\right]+\nabla \left[\frac{\partial T}{{\partial \Sigma }_{other}}\Sigma_{other}\right]+\nabla \left[\frac{\partial T}{\delta W}W\right]\right\},$$

where, for a general nucleation event, A denotes the newly created surface area, which may also be deformed; under equilibrium conditions, it is only created.

### 2.4. Applications of the New Field to Thermodynamic Variables and Functions

Solving the nucleation equation in the continuum-mechanics domain, when scaled to DTA/DSC experimental conditions, requires evaluation of the surface-stress tensor to obtain the surface stress, surface tension, and surface energy [31].

For molecular dynamics (MD) simulations, several surface-stress approximations are available in the literature [36,37].

The present calculation assumes that phase nucleation generates a homogeneous surface stress [37] and that no plastic deformation occurs, because only a solid–liquid transformation is considered. These assumptions are sufficient to compare the model predictions with the experimental data reported in [32,33].

For simplicity, a spherical geometry is adopted and spherical (θ, φ) coordinates are employed. The metric tensor and its contravariant on a 2-sphere are given by

(23) $$g_{ij}=\left(\begin{array}{cc} r^{2} & 0\\ 0 & r^{2}\sin^{2}\theta \end{array}\right),\;\mathrm{and}\;\mathrm{the}\;\mathrm{initial}\;\mathrm{conditions}\;\delta g^{ij}=\left(\begin{array}{cc} r_{0}^{-2} & 0\\ 0 & r_{0}^{-2}\sin^{-2}\theta_{0} \end{array}\right),$$

The surface stress tensor, as presented in [38] and assuming no plastic deformation, is given by

(24a) $$\nabla_{S}\cdot \Sigma =\Theta n+\rho_{0}\ddot{u},$$

(24b) $$\Sigma =\sigma I+2\left(\mu_{0}-\sigma \right)IE+\left(\lambda_{0}-\sigma \right)\left(trE\right)I+\sigma \nabla_{S}u\;\mathrm{on}\;\mathrm{surface}\;S,$$

(24c) $$E=\frac{1}{2}\left(Du+Du^{T}\right),$$

where Θ, E, and u denote the stress, strain, and displacement fields, respectively, and λ and μ are the Lamé coefficients.

#### 2.4.1. The Surface Free Energy

Ferreira recently deduced an equation to calculate the surface energy [31,32,33,36,37,38,39,40] in terms of nucleation vector radius $r_{C}$ and on the stress tensor Σ as follows:

(25) $$\Delta G_{S}={\Delta G}_{S0}\frac{1+r_{o}^{2}R}{1+g^{ij}\cdot \left(g_{ij}⊗R_{ij}\right)}-\int_{r_{0}}^{r_{1}}\Sigma \cdot dr,$$

Considering a 2-sphere, the Ricci scalar gives $R=2/r_{0}^{2}$. The surface energy from state 0 to 1 can be obtained as

(26) $$\Delta G_{S}^{sph}={\Delta G}_{S0}\frac{3}{1+\frac{r^{2}}{r_{0}^{2}}+\frac{r^{2}\sin^{2}\theta }{r_{0}^{2}\sin^{2}\theta_{0}}}-\int_{r_{0}}^{r_{1}}\Sigma \cdot dr,$$

where ${\Delta G}_{S0}=\gamma_{0}$, $r_{0}$, $r_{1}$, Σ, and R are the surface energy in [J·m^−2^], the radius at the thermodynamic state 0 (assumed as equilibrium or initial condition vector radius for Equilibrium, i.e., $\partial \Omega =\delta g_{ij}$) in [m], radius at the state 1 in [m], the surface stress tensor in [N.m^−1^], and the Ricci scalar, respectively.

#### 2.4.2. The Surface Free Energy Potential

The surface free energy potential is found in thermal-field tensor formulation, Γ=A⋅∇T, Equation (22), representing the surface energy potential $\frac{\partial \Delta G_{S}}{\partial r}$ corresponding to one of the terms of the second law of thermodynamics in the present phase-nucleation formulation.

(27) $$\frac{\partial \Delta G_{S}}{\partial r}=\partial_{k}\left[{\Delta G}_{S0}\frac{1+r_{o}^{2}R}{1+g^{ij}\cdot \left(g_{ij}⊗R_{ij}\right)}-\int_{r_{0}}^{r_{1}}\Sigma \cdot dr\right]=-{\Delta G}_{S0}\frac{\left(1+r_{o}^{2}R\right)\left[\partial_{k}g^{ij}\cdot \left(g_{ij}⊗R_{ij}\right)+g^{ij}\cdot \left(g_{ij;k}⊗R_{ij}\right)+g^{ij}\cdot \left(g_{ij}⊗R_{ij;k}\right)\right]}{{\left[1+g^{ij}\cdot \left(g_{ij}⊗R_{ij}\right)\right]}^{2}}-\Sigma ,$$

For a two-dimensional spherical surface of radius $r_{0}$, the Ricci scalar curvature reads $R=\frac{2}{r_{0}^{2}}$.

Using this curvature, the surface free energy potential associated with the transition from state 0 to state 1 can be expressed as

(28) $${\left.\frac{\partial \Delta G_{S}}{\partial r}\right|}^{sph}={\Delta G}_{S0}\frac{-6\left[\frac{r\sin^{2}\theta }{r_{0}^{2}\sin^{2}\theta_{0}}+\frac{r}{r_{0}^{2}}\right]}{{\left(1+\frac{r^{2}}{r_{0}^{2}}+\frac{r^{2}\sin^{2}\theta }{r_{0}^{2}\sin^{2}\theta_{0}}\right)}^{2}}-\Sigma ,$$

where ${\Delta G}_{S0}=\gamma_{0}$, $r_{0}$, Σ, and R are the surface energy in [J·m^−2^]; the radius at the thermodynamic state 0 (assumed as equilibrium or initial condition vector radius for Equilibrium, i.e., $\partial \Omega =\delta g_{ij}$) in [m], the surface stress tensor in [N·m^−1^], and the Ricci scalar, respectively.

#### 2.4.3. The Surface Entropy

The surface entropy can be readily derived from the surface free energy, Equation (25), as follows:

(29) $$\Delta S_{S}=\frac{{\Delta G}_{S0}}{\Delta T}\frac{1+r_{o}^{2}R}{1+g^{ij}\cdot \left(g_{ij}⊗R_{ij}\right)}-\frac{1}{\Delta T}\int_{r_{0}}^{r_{1}}\Sigma \cdot dr,$$

Considering a 2-sphere, the Ricci scalar $R=2/r_{0}^{2}$ gives

(30) $$\Delta S_{S}^{sph}=\frac{{\Delta G}_{S0}}{\Delta T}\frac{3}{1+\frac{r^{2}}{r_{0}^{2}}+\frac{r^{2}\sin^{2}\theta }{r_{0}^{2}\sin^{2}\theta_{0}}}-\frac{1}{\Delta T}\int_{r_{0}}^{r_{1}}\Sigma \cdot dr,$$

where ${\Delta G}_{S0}=\gamma_{0}$, $r_{0}$, ΔT, Σ, and R are the surface energy in [J·m^−2^], the radius at the thermodynamic state 0 (assumed as equilibrium or initial condition vector radius for Equilibrium, i.e., $\partial \Omega =\delta g_{ij}$) in [m], the undercooling in [K], the surface stress tensor in [N·m^−1^], and the Ricci scalar, respectively.

#### 2.4.4. The Surface Entropy Potential

The surface entropy potential is deduced from Equation (29) as

(31) $$\frac{\partial \Delta S_{S}}{\partial r}=-\frac{{\Delta G}_{S0}}{\Delta T}\frac{\left(1+r_{o}^{2}R\right)\left[\partial_{k}g^{ij}\cdot \left(g_{ij}⊗R_{ij}\right)+g^{ij}\cdot \left(g_{ij;k}⊗R_{ij}\right)+g^{ij}\cdot \left(g_{ij}⊗R_{ij;k}\right)\right]}{{\left[1+g^{ij}\cdot \left(g_{ij}⊗R_{ij}\right)\right]}^{2}}-\frac{1}{\Delta T}\Sigma ,$$

Using the standard spherical coordinates on a 2-sphere of radius $r_{0}$, the Ricci scalar evaluates to $R=\frac{2}{r_{0}^{2}}$. Substituting this expression for R into Equation (31) yields

(32) $${\left.\frac{\partial \Delta S_{S}}{\partial r}\right|}^{sph}=\frac{{\Delta G}_{S0}}{\Delta T}\frac{-6\left[\frac{r\sin^{2}\theta }{r_{0}^{2}\sin^{2}\theta_{0}}+\frac{r}{r_{0}^{2}}\right]}{{\left(1+\frac{r^{2}}{r_{0}^{2}}+\frac{r^{2}\sin^{2}\theta }{r_{0}^{2}\sin^{2}\theta_{0}}\right)}^{2}}-\frac{1}{\Delta T}\Sigma ,$$

where ${\Delta G}_{S0}=\gamma_{0}$, $r_{0}$, ΔT, Σ, and R are the surface energy in [J·m^−2^], the radius at the thermodynamic state 0 (assumed as equilibrium or initial condition vector radius for Equilibrium, i.e., $\partial \Omega =\delta g_{ij}$) in [m], the undercooling in [K], the surface stress tensor in [N·m^−1^], and the Ricci scalar, respectively.

#### 2.4.5. New Nucleation Formulation Against Experimental Data

The present formulation, expressed in terms of the primitive thermodynamic variables [31], has recently been tested against experimental data for two alloy compositions reported in [32,33]; the results are not reproduced here. In Figure 2 (adapted from [33]), the nucleation rate Equation (21) is plotted to illustrate the physical variables that most strongly influence its calculation. The basic idea of the experimental procedures described in Refs. [32,33] concerns the nature of the Gibbs–Thomson coefficient, Γ. When only temperature varies—i.e., in the absence of compositional gradients, mechanical deformation, etc.—Γ reduces to a temperature-only form of the thermal-field tensor, which we denote as Γ=Γ(∇T). Consequently, if the Gibbs–Thomson coefficient is a function of the local temperature gradient ∇*T*, the hypothesis is verified. This is precisely what is observed in references [32,33] for the first-order approximation and second-order exact nucleation formulation.

## 3. Applications

### 3.1. Quasicrystalline Microstructures

The formation of a quasiperiodic crystal, such as that reported by Bindi and Parisi [41] and illustrated in Figure 3, can be interpreted within the present thermodynamic framework as the consequence of a continuous evolution of the configurational free energy, $\Delta G_{C}$. Variations in $\Delta G_{C}$ continuously modify the equilibrium contact angle, *θ*, driving the system toward the minimum of the total critical Gibbs free energy. As a result, the growth front continuously adjusts its crystallographic orientation and interfacial configuration, producing a quasiperiodic structure rather than a conventional periodic crystal. In this sense, a quasicrystal may be viewed as a continuously evolving monocrystal, whose growth direction and local atomic arrangement are continuously optimized according to the thermodynamic equilibrium condition expressed by the following equation:

(33) $$\Gamma =A\cdot \nabla T=-\frac{3}{4}\frac{\left[\left(\Delta S_{V}\Delta T+\frac{{\partial \gamma }_{SL}}{\partial r}\right)f\left(\theta \right)+\gamma_{SL}\frac{\partial f\left(\theta \right)}{\partial r}\right]}{\frac{1}{\Delta T}\left[\frac{\partial \left(\Delta S_{V}\Delta Tf\left(\theta \right)\right)}{\partial r}\right]}=-\frac{3}{4}\frac{\left[\Delta G_{V}+\frac{\partial \Delta G_{S}}{\partial r}+\Delta G_{C}\right]}{\frac{\partial \Delta S_{V}}{\partial r}},$$

### 3.2. A New Butler’s Formulation for Thermodynamic Equilibrium Shift

Butler’s original thermodynamic formulation of surfaces [42] does not include the substrate contribution. When the system departs from equilibrium, the surface free energy rises with increasing thermal gradients. Because the substrate contribution is omitted, the calculated surface energy deviates from the true value; consequently, reliable predictions are limited to conditions close to equilibrium.

Considering the bulk free energy ${\bar{G}}^{B}$ [J/m^3^], the surface free energy ${\bar{G}}^{S}$ [J/m^3^], and the configurational free energy ${\bar{G}}^{C}$ [J/m^3^] within Butler’s classical formulation yields the following set of expressions:

(34a) $$\gamma =\gamma_{SV}+\frac{RT}{A_{SV}}ln\left(\frac{1-\sum_{i=1}^{n}X_{SLi}^{S}}{1-\sum_{i=1}^{n}X_{SLi}^{B}}\right)+\frac{1}{A_{SV}}\left[{\bar{G}}_{SV}^{E,S}\left(T,X_{SL1}^{S},\ldots ,X_{SLn}^{S}\right)-{\bar{G}}_{SV}^{E,B}\left(T,X_{SL1}^{B},\ldots ,X_{SLn}^{B}\right)-{\bar{G}}_{SV}^{E,C}\left(T,X_{SL1}^{S},\ldots ,X_{SLn}^{S}\right)\right],$$

(34b) $$\gamma =\gamma_{SL1}+\frac{RT}{A_{SL1}}ln\left(\frac{X_{SL1}^{S}}{X_{SL1}^{B}}\right)+\frac{1}{A_{SL1}}\left[{\bar{G}}_{SL1}^{E,S}\left(T,X_{SL1}^{S},\ldots ,X_{SLn}^{S}\right)-{\bar{G}}_{SL1}^{E,B}\left(T,X_{SL1}^{B},\ldots ,X_{SLn}^{B}\right)-{\bar{G}}_{SL1}^{E,C}\left(T,X_{SL1}^{S},\ldots ,X_{SLn}^{S}\right)\right]$$

(34c) $$\gamma =\gamma_{SLn}+\frac{RT}{A_{SLn}}ln\left(\frac{X_{SLn}^{S}}{X_{SLn}^{B}}\right)+\frac{1}{A_{SLn}}\left[{\bar{G}}_{SLn}^{E,S}\left(T,X_{SL1}^{S},\ldots ,X_{SLn}^{S}\right)-{\bar{G}}_{SLn}^{E,B}\left(T,X_{SL1}^{B},\ldots ,X_{SLn}^{B}\right)-{\bar{G}}_{SLn}^{E,C}\left(T,X_{SL1}^{S},\ldots ,X_{SLn}^{S}\right)\right]$$

The surface free energy potential can also be considered through the following set of equations:

(35a) $$\frac{\partial \gamma }{\partial r}=\frac{{\partial \gamma }_{SV}}{\partial r}-\frac{RT}{A_{SV}^{2}}\frac{{\partial A}_{SV}}{\partial r}ln\left(\frac{1-\sum_{i=1}^{n}X_{SLi}^{S}}{1-\sum_{i=1}^{n}X_{SLi}^{B}}\right)-\frac{RT}{A_{SV}}\left(\frac{\sum_{i=1}^{n}\frac{{\partial X}_{SLi}^{S}}{\partial r}}{1-\sum_{i=1}^{n}X_{SLi}^{S}}\right)\;-\frac{1}{A_{SV}^{2}}\frac{{\partial A}_{SV}}{\partial r}\left[{\bar{G}}_{SV}^{E,S}\left(T,X_{SL1}^{S},\ldots ,X_{SLn}^{S}\right)-{\bar{G}}_{SV}^{E,B}\left(T,X_{SL1}^{B},\ldots ,X_{SLn}^{B}\right)-{\bar{G}}_{SV}^{E,C}\left(T,X_{SL1}^{S},\ldots ,X_{SLn}^{S}\right)\right]\;\;+\frac{1}{A_{SV}}\left[\frac{\partial {\bar{G}}_{SV}^{E,S}\left(T,X_{SL1}^{S},\ldots ,X_{SLn}^{S}\right)}{\partial r}-\frac{\partial {\bar{G}}_{SV}^{E,B}\left(T,X_{SL1}^{B},\ldots ,X_{SLn}^{B}\right)}{\partial r}-\frac{\partial {\bar{G}}_{SV}^{E,C}\left(T,X_{SL1}^{S},\ldots ,X_{SLn}^{S}\right)}{\partial r}\right],$$

(35b) $$\frac{\partial \gamma }{\partial r}=\frac{{\partial \gamma }_{SL1}}{\partial r}-\frac{RT}{A_{SL1}^{2}}\frac{{\partial A}_{SL1}}{\partial r}ln\left(\frac{X_{SL1}^{S}}{X_{SL1}^{B}}\right)-\frac{RT}{A_{SL1}}\left(\frac{\frac{{\partial X}_{SL1}^{S}}{\partial r}}{X_{SL1}^{S}}\right)\;-\frac{1}{A_{SL1}^{2}}\frac{{\partial A}_{SL1}}{\partial r}\left[{\bar{G}}_{SL1}^{E,S}\left(T,X_{SL1}^{S},\ldots ,X_{SLn}^{S}\right)-{\bar{G}}_{SL1}^{E,B}\left(T,X_{SL1}^{B},\ldots ,X_{SLn}^{B}\right)-{\bar{G}}_{SL1}^{E,C}\left(T,X_{SL1}^{S},\ldots ,X_{SLn}^{S}\right)\right]\;+\frac{1}{A_{SL1}}\left[\frac{\partial {\bar{G}}_{SL1}^{E,S}\left(T,X_{SL1}^{S},\ldots ,X_{SLn}^{S}\right)}{\partial r}-\frac{\partial {\bar{G}}_{SL1}^{E,B}\left(T,X_{SL1}^{B},\ldots ,X_{SLn}^{B}\right)}{\partial r}-\frac{\partial {\bar{G}}_{SL1}^{E,C}\left(T,X_{SL1}^{S},\ldots ,X_{SLn}^{S}\right)}{\partial r}\right]$$

(35c) $$\frac{\partial \gamma }{\partial r}=\frac{{\partial \gamma }_{SLn}}{\partial r}-\frac{RT}{A_{SLn}^{2}}\frac{{\partial A}_{SLn}}{\partial r}ln\left(\frac{X_{SLn}^{S}}{X_{SLn}^{B}}\right)-\frac{RT}{A_{SLn}}\left(\frac{\frac{{\partial X}_{SLn}^{S}}{\partial r}}{X_{SLn}^{S}}\right)\;-\frac{1}{A_{SLn}^{2}}\frac{{\partial A}_{SLn}}{\partial r}\left[{\bar{G}}_{SLn}^{E,S}\left(T,X_{SL1}^{S},\ldots ,X_{SLn}^{S}\right)-{\bar{G}}_{SLn}^{E,B}\left(T,X_{SL1}^{B},\ldots ,X_{SLn}^{B}\right)-{\bar{G}}_{SLn}^{E,C}\left(T,X_{SL1}^{S},\ldots ,X_{SLn}^{S}\right)\right]\;+\frac{1}{A_{SLn}}\left[\frac{\partial {\bar{G}}_{SLn}^{E,S}\left(T,X_{SL1}^{S},\ldots ,X_{SLn}^{S}\right)}{\partial r}-\frac{\partial {\bar{G}}_{SLn}^{E,B}\left(T,X_{SL1}^{B},\ldots ,X_{SLn}^{B}\right)}{\partial r}-\frac{\partial {\bar{G}}_{SLn}^{E,C}\left(T,X_{SL1}^{S},\ldots ,X_{SLn}^{S}\right)}{\partial r}\right]$$

The configurational free energy can be explained as follows. Vitreloy 1 (41.2 wt% Zr, 13.8 wt% Ti, 12.5 wt% Cu, 10.0 wt% Ni, 22.5 wt% Be) is a prototypical bulk metallic glass whose thermodynamics are dominated by the configurational contribution to the free energy. Zirconium behaves as a high-packing component (HPC); however, when the composition is brought to that of Vitreloy 1, the alloy remains amorphous—no phase transformation occurs and no latent heat of crystallization is released. Thus, adding solutes shifts the liquid–solid equilibrium, stabilizing the liquid over a wider temperature range. This stabilization originates from severe atomic-size mismatch; the crystalline unit cell can no longer achieve close packing, producing a geometric configuration that suppresses crystallization.

### 3.3. Climate Change Scenario

The mechanisms underlying global warming and climate inversion can be associated with the following processes. In a black body experiment, an incident beam of light enters through an aperture and gradually loses energy to the black body through successive reflections, eventually emerging from the aperture with low energy; by analogy, greenhouse gases act in Earth’s atmosphere, over the continents and oceans, raising the global temperature [43,44].

A key problem that emerges is the solubility of CO_2_, which declines as the temperature of the ocean surface rises, preventing effective capture and creating a high-temperature, low-solubility barrier that hinders the gas from moving into deeper waters. In these deeper layers, high pressure continuously increases CO_2_ solubility with depth.

When this capture mechanism is interrupted or becomes inefficient, CO_2_ remains in the atmosphere, heating the oceans, until excessive melting of the polar ice caps disrupts the Atlantic Meridional Overturning Circulation (AMOC) and other ocean currents. This disruption triggers a climate inversion process that cools the oceans and thereby restores CO_2_ solubility.

The new general nucleation thermodynamic theory not only reveals the physical nature of the thermal field gradient but also contributes to the understanding of climate change by elucidating non-equilibrium water-phase cycle transformations and by establishing the minimum and maximum heat transfer coefficients for dry and moist air [45].

Using the thermodynamic properties of hexagonal ice, the simulations presented below employ a recently derived solution for the transient solidification of pure and eutectic materials under convective boundary conditions.

The model was used to predict the thermal gradient [45] in the liquid phase for a series of humid-air conditions, characterized by the following Biot numbers: {0.12605, 0.252101, 0.378151, 0.630252, 5.04202}. The similarity variable φ can be calculated from the following equation:

(36) φ=SteS ψs,φBiot2φexpBiot+Biot24 φ2erfcφ+Biot2φ+1π expφ2−1π expφ+Biot2φ2expBiot+Biot24 φ2+SteL N nπ erfcnφ exp(n2φ2)+Ω*

where $Ste=\frac{C_{PS}\left(T_{F}-T_{\infty }\right)}{L}$, ${Ste}_{L}=\frac{C_{PL}\left(T_{P}-T_{F}\right)}{L}$ are the Stefan numbers for the solid and liquid phases and, $\Omega^{*}=\frac{Biot}{\varphi }$ is the dimensionless velocity $\Omega =\frac{2h\alpha_{S}}{k_{S}}$, the coefficients $N=\frac{\alpha_{L}\rho_{L}}{\alpha_{S}\rho_{S}}$ and $n=\sqrt{\frac{\alpha_{S}}{\alpha_{L}}}\;$.

The thermal gradient of the second phase can be calculated as

(37) $${\left.\frac{\partial T_{L}}{\partial x}\right|}_{x=s^{+}}=-\frac{2\varphi \;n}{\sqrt{\pi }\;s}\cdot \frac{\left(T_{P}-T_{F}\right)}{\mathrm{erfc}\left(n\varphi \right)}\cdot \exp\left(-n^{2}\varphi^{2}\right)$$

## 4. Results and Discussion

The aim of the present work is to demonstrate the effect of the thermal gradient on the bulk free energy $\Delta G_{V}$, surface free energy $\Delta G_{S}$, and configurational free energy $\Delta G_{C}$. We considered the α (FCC_A1) phase based on the alloy composition Al-3 wt%Cu-0.5 wt%Mg-0.11 wt%Cu, whose equilibrium data were calculated using Thermo-Calc with the aluminum database TTAl7.

The equilibrium shift was calculated by imposing a thermal gradient and solving Equation (17) in primitive thermodynamic variables, as in [31], to obtain the nucleation radius for a given thermal gradient. In the present calculation, only the thermal and solutal components were considered. The theoretical results for the FCC_A1 phase of the alloy Al-3 wt%Cu, 0.5 wt%Mg, 0.11 wt%Cu are summarized in Table 1 and Table 2 as a function of the thermal gradient. The corresponding data shown in bold in Table 1 and Table 2 represent the experimental results obtained for the thermal gradients reported in Ref. [33].

Figure 4 shows the bulk free energy of the FCC_A1 phase as a function of the thermal gradient. It is observed that $\Delta G_{V}$ decreases as the thermal gradient increases. In the present study, the bulk free energy values are negative. The nucleation radius also decreases as the thermal gradient increases, as confirmed in Table 1.

Figure 5 shows the surface free energy of the FCC_A1 phase as a function of the thermal gradient. It is observed that $\Delta G_{S}$ increases as the thermal gradient increases. In this study, the surface free energy values are positive.

Figure 6 presents the configurational free energy of the FCC_A1 phase as a function of the thermal gradient. It is observed that $\Delta G_{C}$ decreases as the thermal gradient increases, similarly to the bulk free energy $\Delta G_{V}$. This behavior is expected, as $\Delta G_{C}$ arises from the substrate energy contribution and is associated with minimizing the total critical free energy $\Delta G=\Delta G_{V}+\frac{\partial \Delta G_{S}}{\partial r}+\Delta G_{C}$.

Figure 7 shows the bulk entropy of the FCC_A1 phase as a function of the thermal gradient. It is observed that $\Delta S_{V}$ decreases as the thermal gradient increases, just as the bulk free energy does.

Figure 8 shows the surface entropy of the FCC_A1 phase plotted against the thermal gradient. It is observed that $\Delta S_{S}$ increases monotonically as the thermal gradient rises, mirroring the behavior observed for the surface free energy.

Figure 9 presents the configurational entropy of the FCC_A1 phase as a function of the thermal gradient. It is observed that $\Delta S_{C}$ increases slowly as the thermal gradient increases. This behavior is expected, as $\Delta G_{C}$ arises from the substrate energy contribution and is associated with minimizing the total critical entropy $\Delta S=\Delta S_{V}+\frac{\partial \Delta S_{S}}{\partial r}+\Delta S_{C}$, as observed in Equation (20) for the first-order critical-radius approximation.

Evidence of a thermodynamic equilibrium shift can be observed through the dependence of the Gibbs–Thomson coefficient, the so-called thermal field tensor, Γ, on the thermal field gradient T, as predicted by the first law of thermodynamics [31,32,33] and shown in Figure 10.

Figure 11 presents the theoretical prediction of the equilibrium-shift chemical potential as a function of (A) the thermal gradient and (B) the undercooling. At low thermal gradients and low undercooling, the equilibrium chemical potential is expected to be approximately −5 × 10^5^ J mol^−1^.

Climate inversion can be tracked by simultaneously solving the nucleation-rate equation (as a function of the temperature gradient) and the transient solidification problem, which yields temperature gradients for a range of superheating levels. Table 3 and Table 4 report the thermophysical properties calculated for water vapor–hexagonal ice equilibrium-shift transformations as a function of the thermal gradient. In Figure 12, the generalized Gibbs–Thomson coefficient, **Γ** (the thermal-field tensor), for the water vapor–hexagonal ice transformation is plotted as a function of the imposed thermal gradient. As shown, **Γ** increases with the thermal gradient for both the first-order (linear) approximation and the second-order closed-form nucleation formulation.

Figure 13 illustrates moist-air superheating from 1.6 K to 2.0 K; a gradient of 1530.6 K m^−1^ highlighted in bold in Table 3 marks the climate inversion point. Note that for high Biot numbers [45], conditions that occur in certain planetary regions, especially temperate zones, the effect manifests as a partial inversion that leads to a severe climatic response.

## 5. Conclusions

The following conclusions can be drawn from the results and discussion presented in this paper:

- The nucleation formulation previously presented in terms of fundamental thermodynamic variables [31] is now expressed as a function of the relevant free energies.
- The analyzed nucleation process exhibits quadratic behavior with respect to the nucleation radius.
- The bulk and configurational free energies exhibit signs opposite to that of the surface free energy.
- Because the substrate contribution tends to minimize the local free energy, the configurational and bulk free energies share the same sign and trend.
- A new Butler-type formulation is derived to account for the substrate interfacial energy.
- The non-periodicity of quasicrystalline structures can be attributed to a continuous change in the contact angle, thereby minimizing the critical free energy barrier.

## Figures and Tables

**Figure 1:**
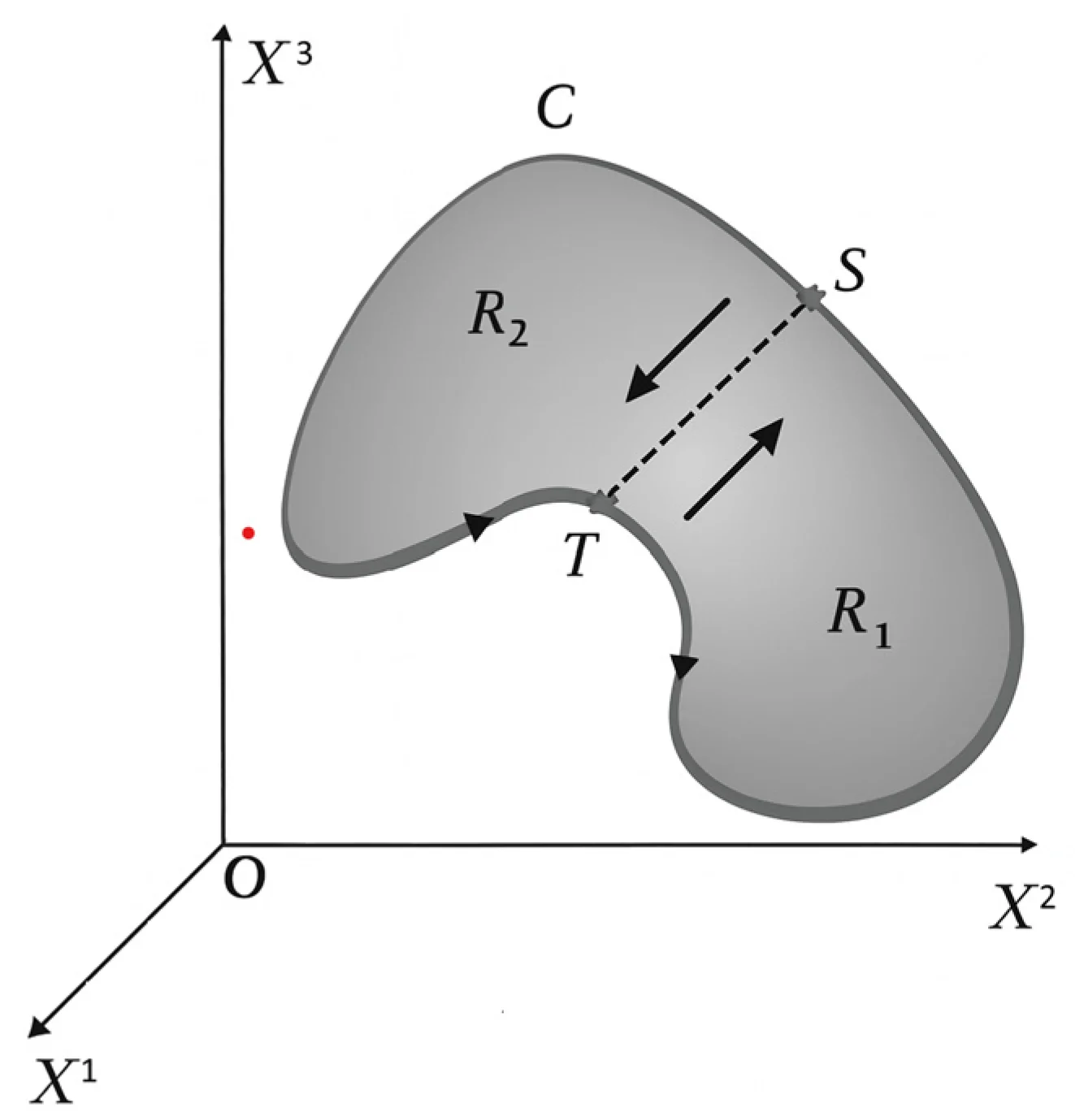
Schematic representation of the heat and work path following the curve TS

**Figure 2:**
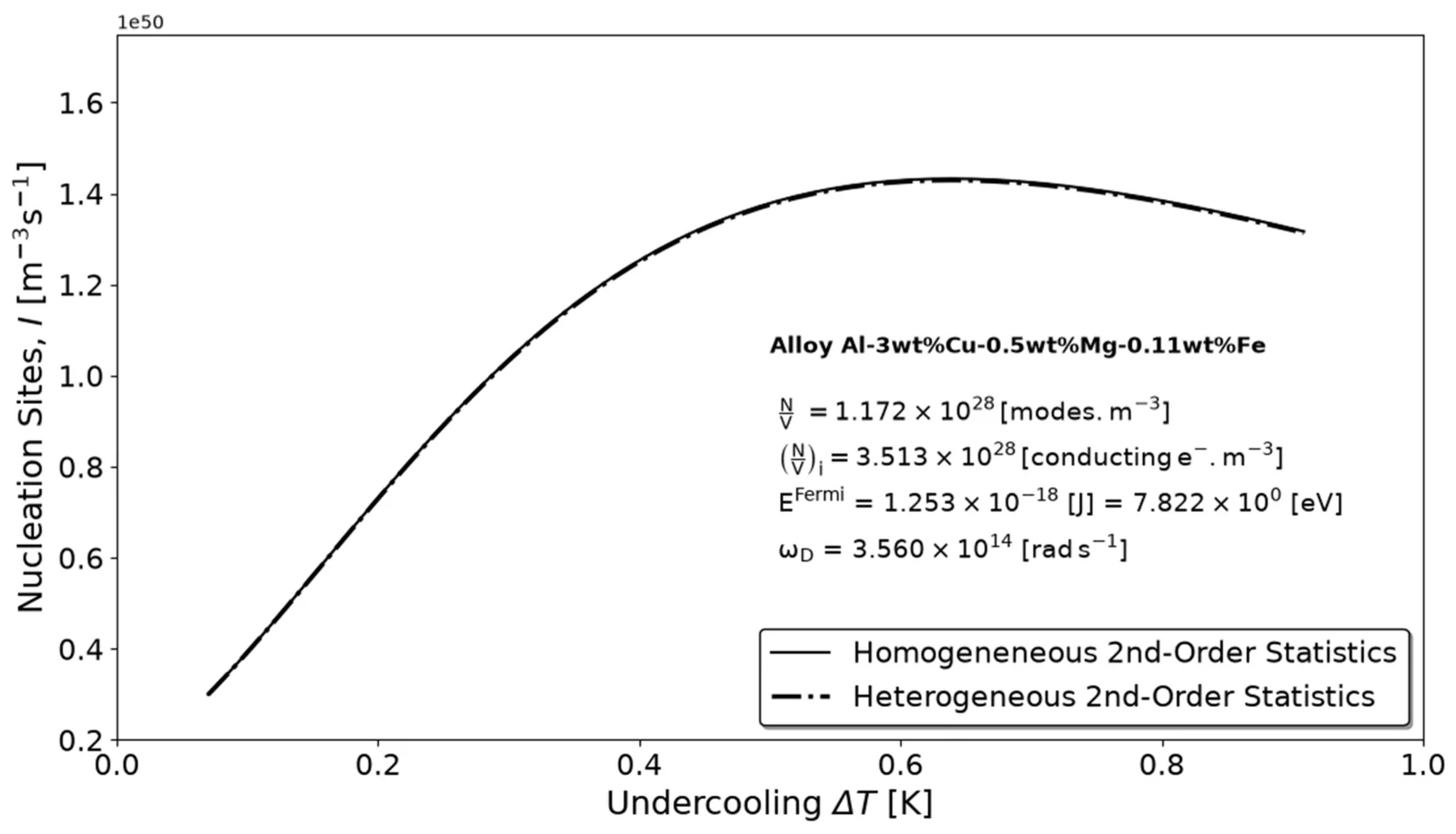
Nucleation rate as a function of undercooling for the FCC_A1 phase of the alloy Al-3 wt%Cu-0.5 wt%Mg-0.11 wt%Fe.

**Figure 3:**
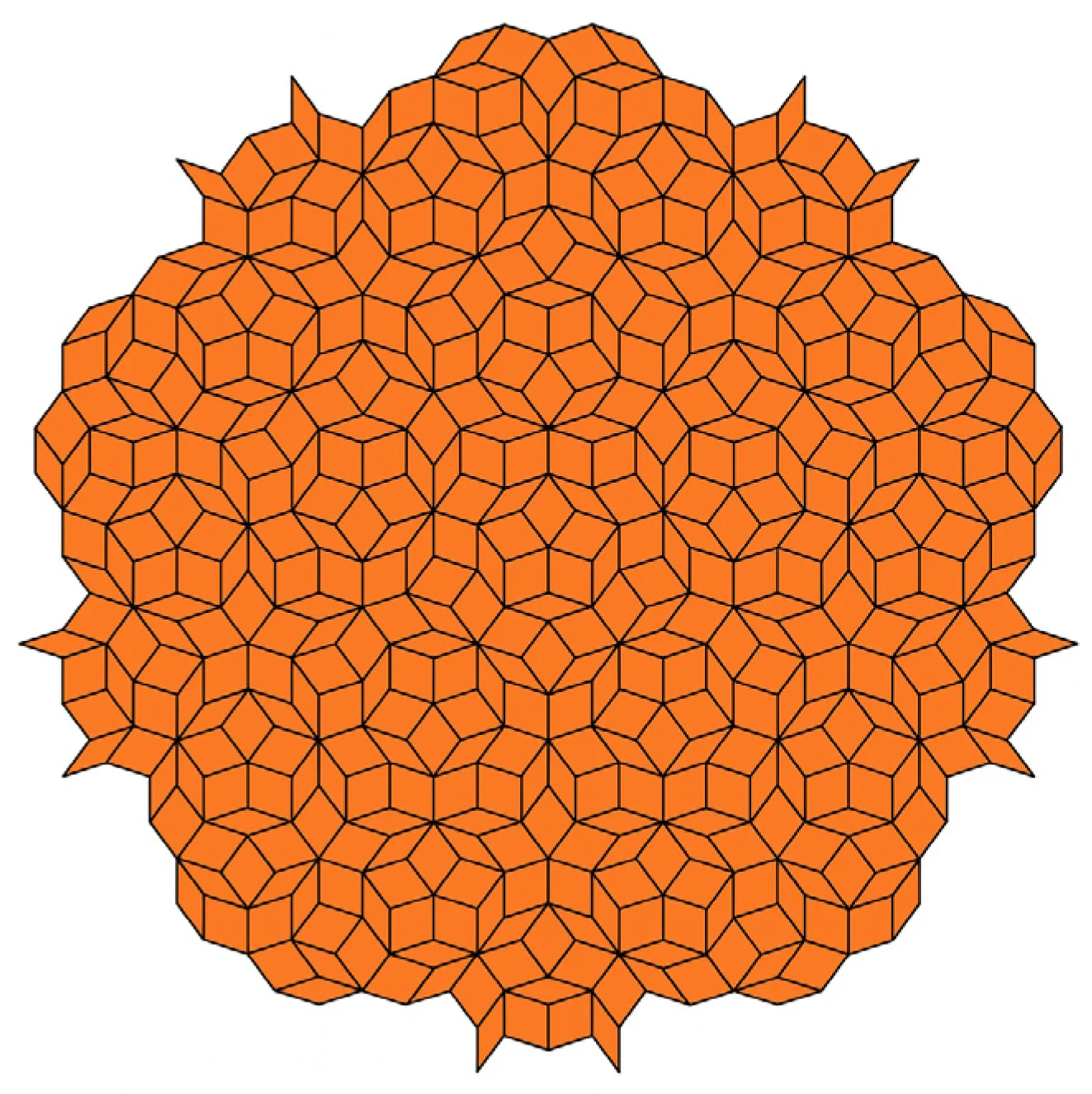
Quasicrystalline microstructure showing continuous changes in the nucleation angle, adapted from Bindi and Parisi [41].

**Figure 4:**
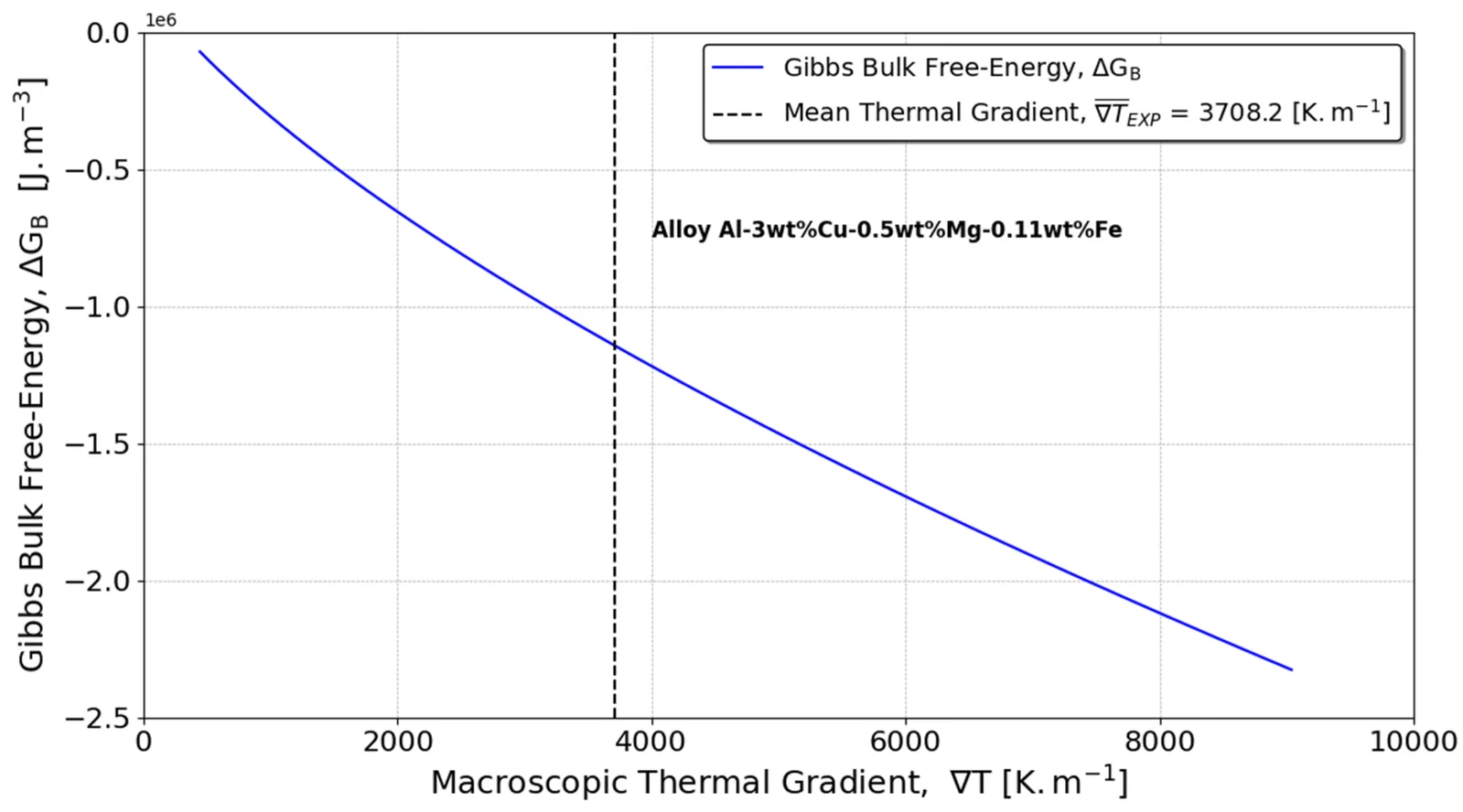
Gibbs bulk free energy as a function of the thermal gradient.

**Figure 5:**
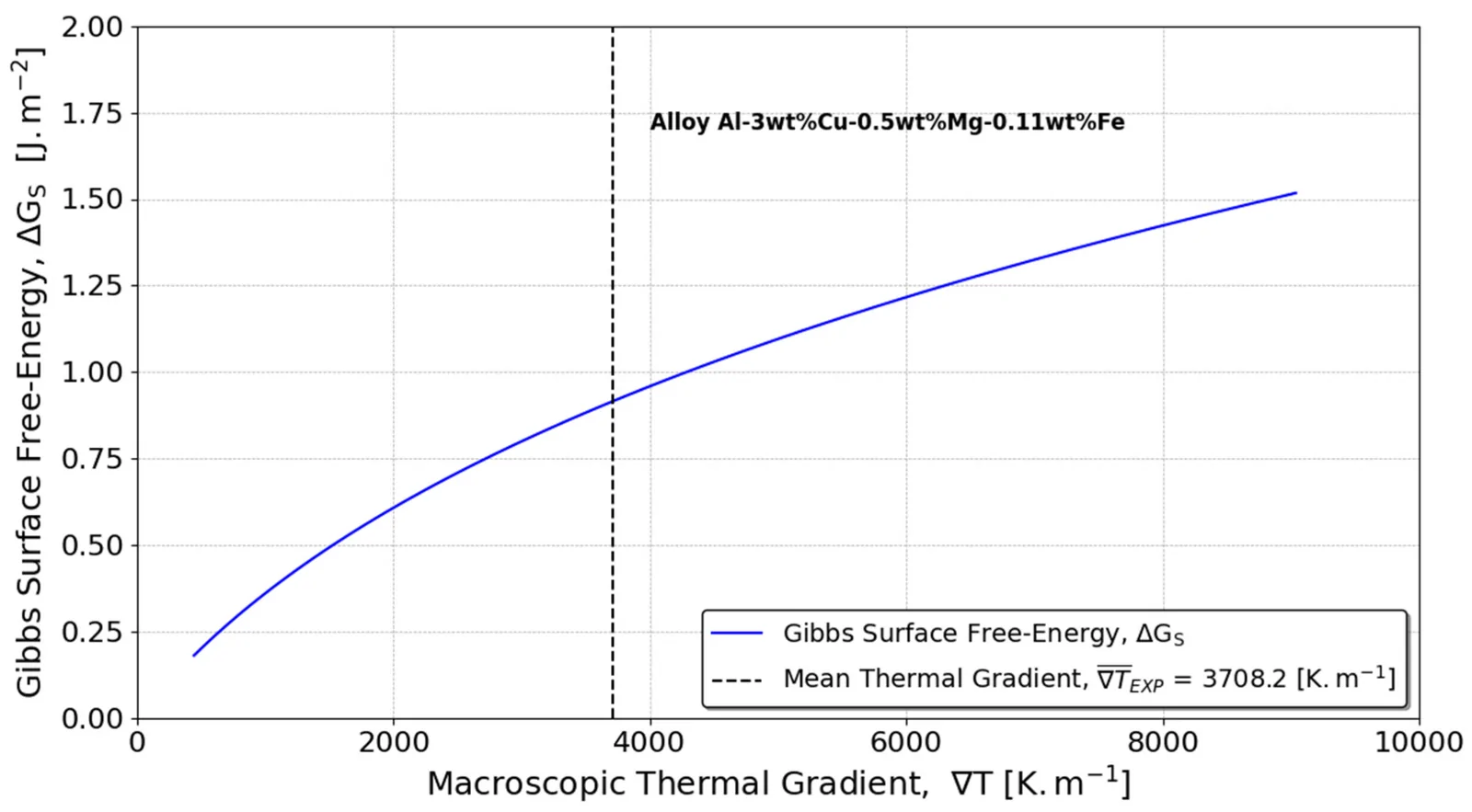
Gibbs surface free energy as a function of the thermal gradient.

**Figure 6:**
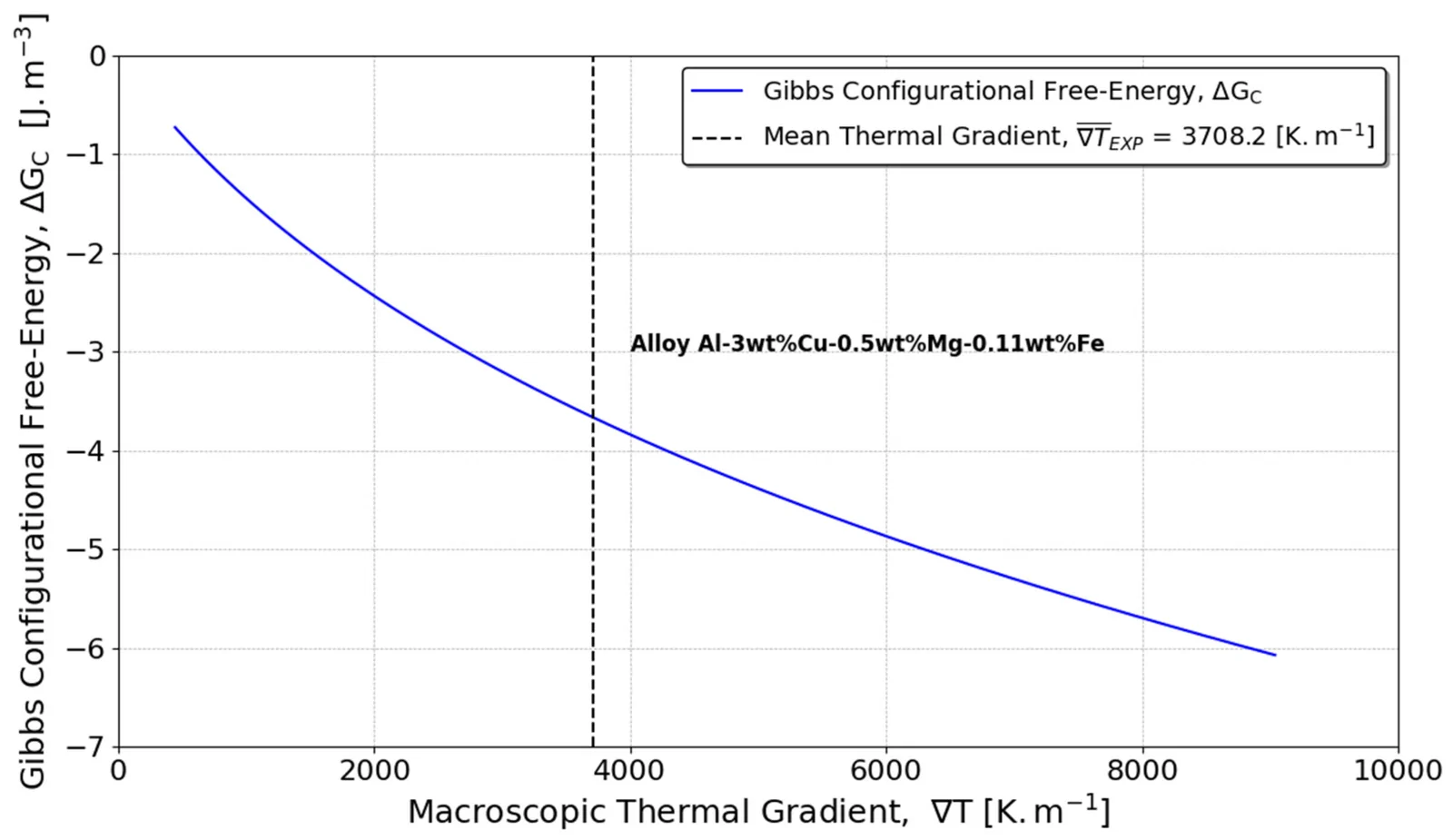
Configurational free energy as a function of the thermal gradient.

**Figure 7:**
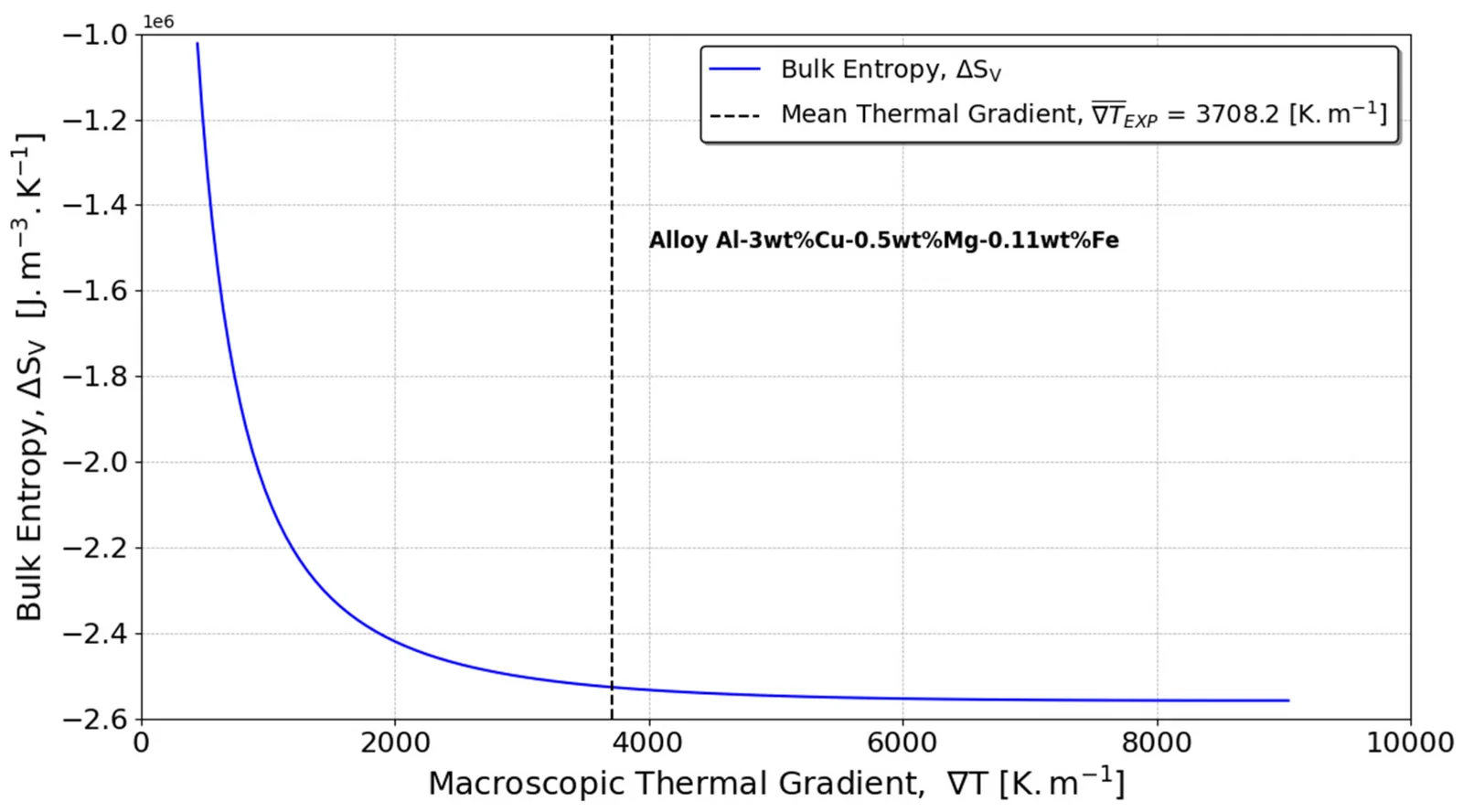
Bulk entropy as a function of the thermal gradient.

**Figure 8:**
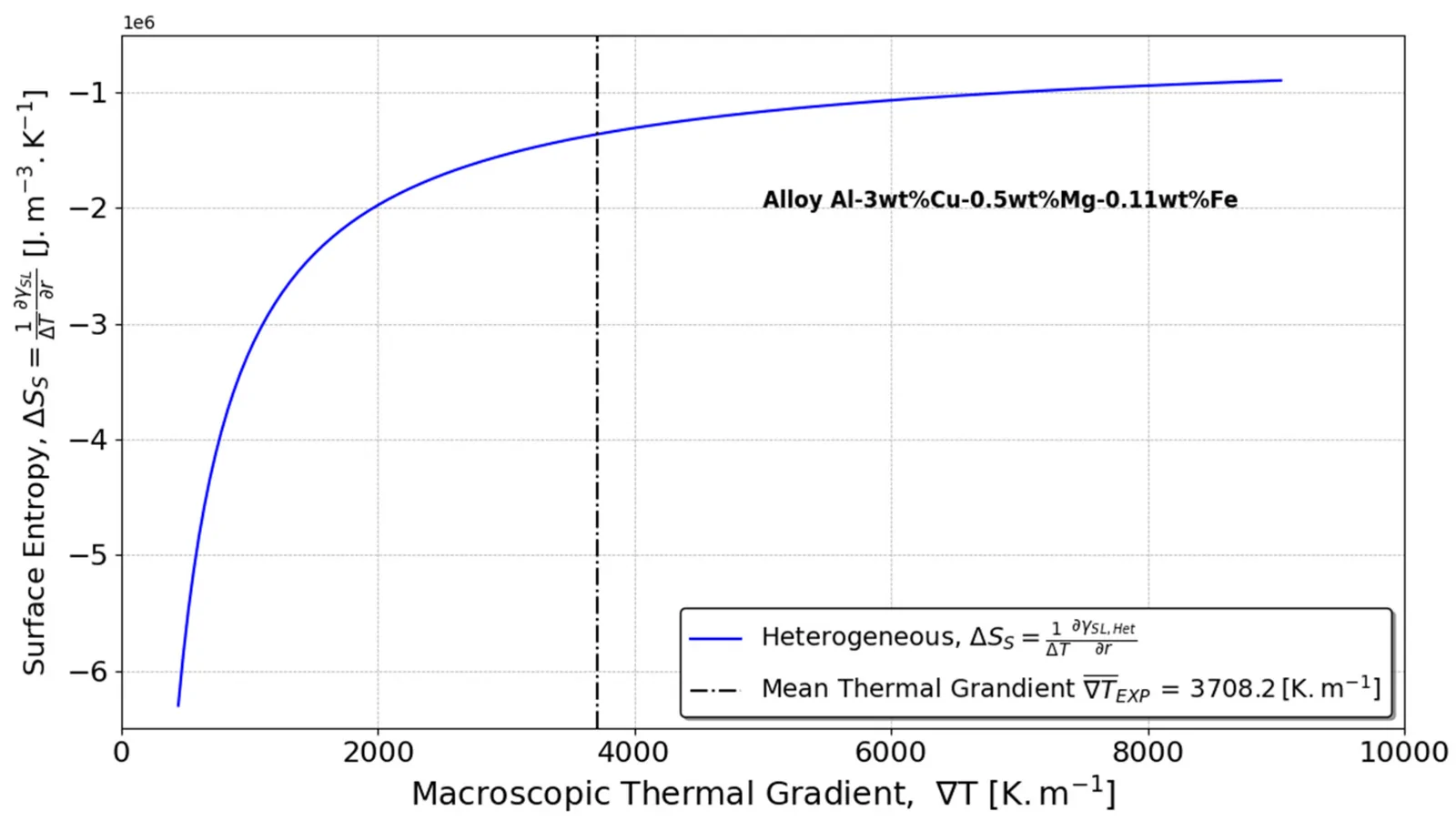
Surface entropy as a function of the thermal gradient.

**Figure 9:**
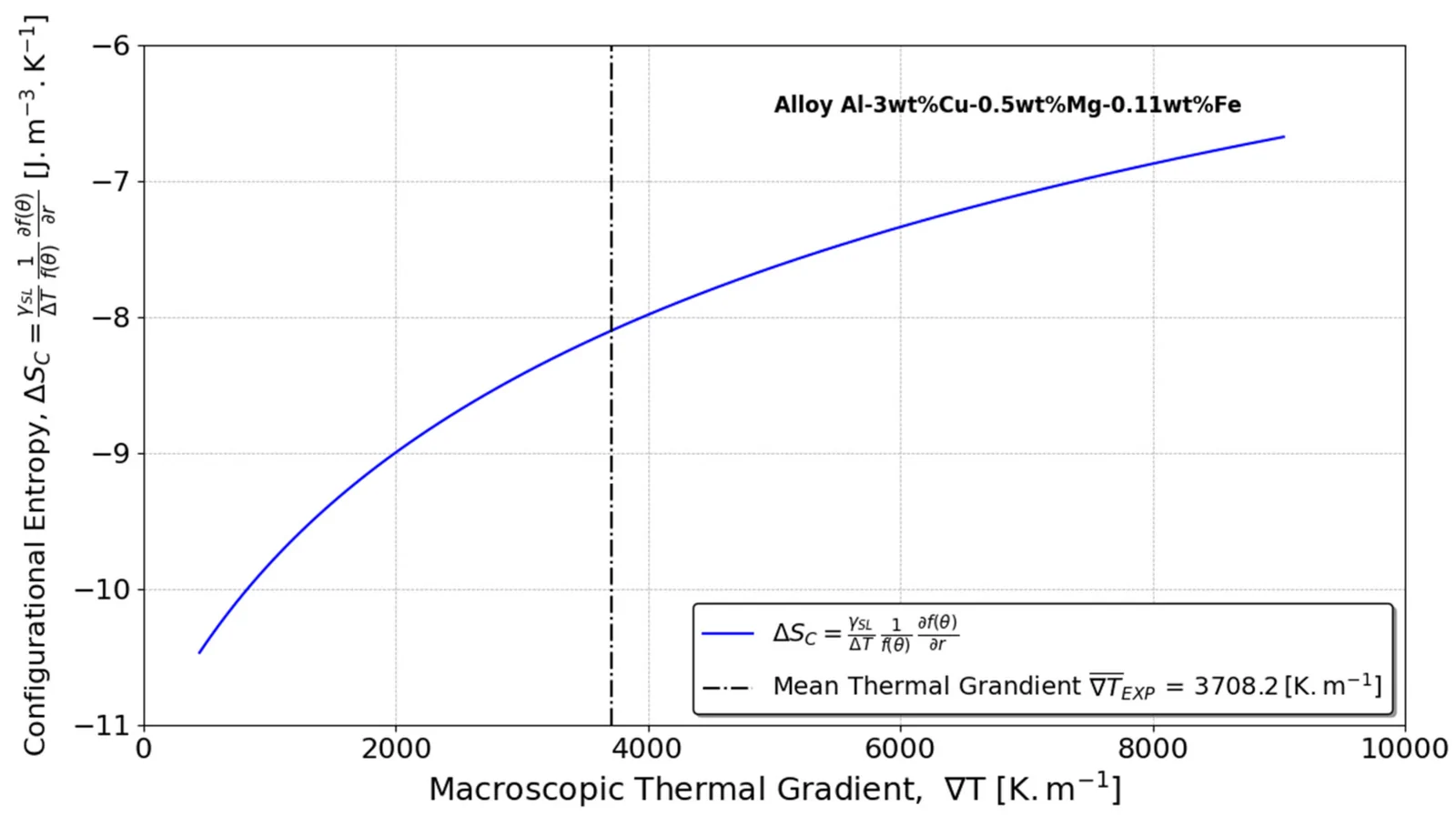
Configurational entropy as a function of the thermal gradient.

**Figure 10:**
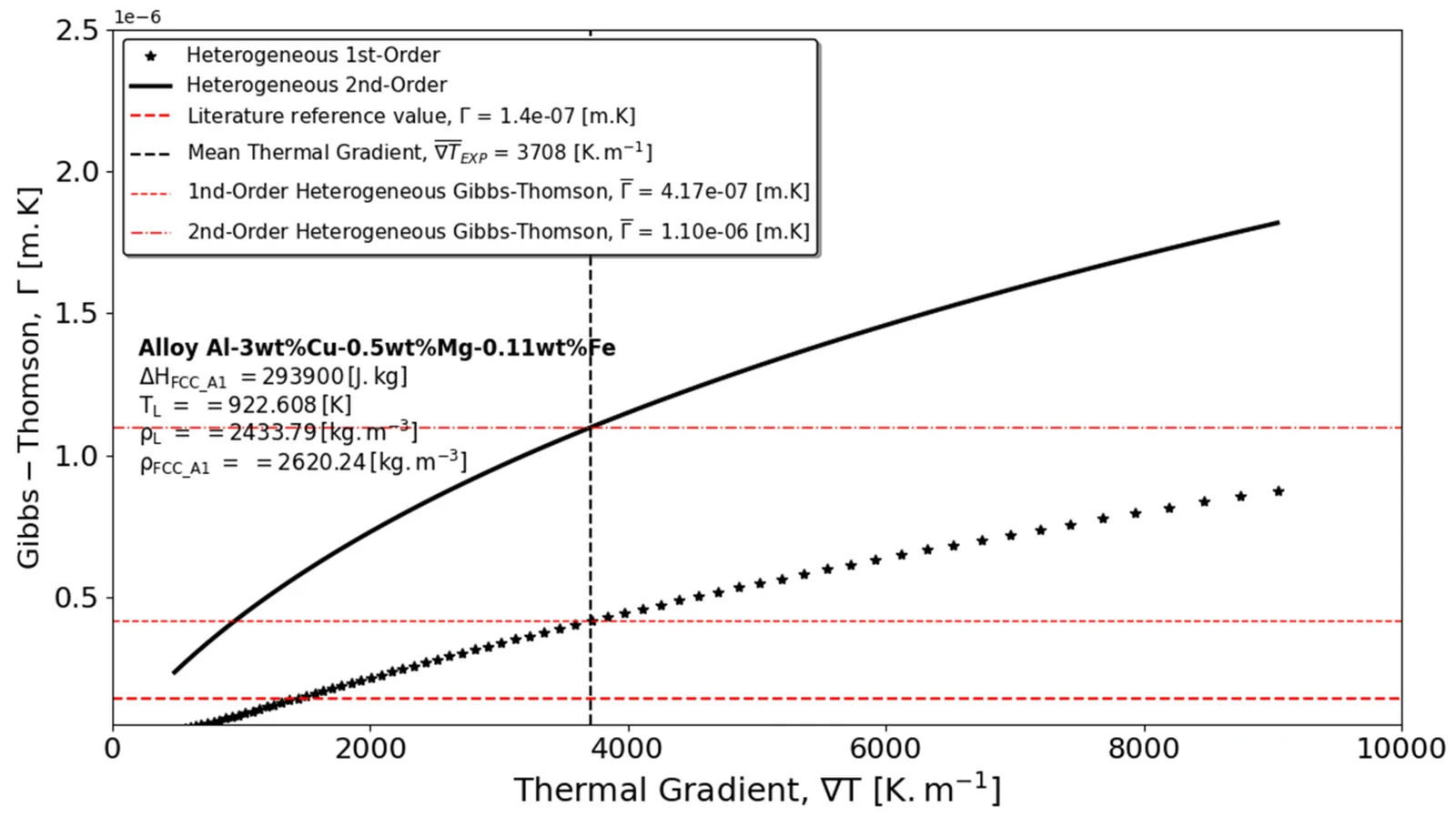
Thermal field tensor (Gibbs–Thomson coefficient) as a function of the thermal gradient.

**Figure 11:**
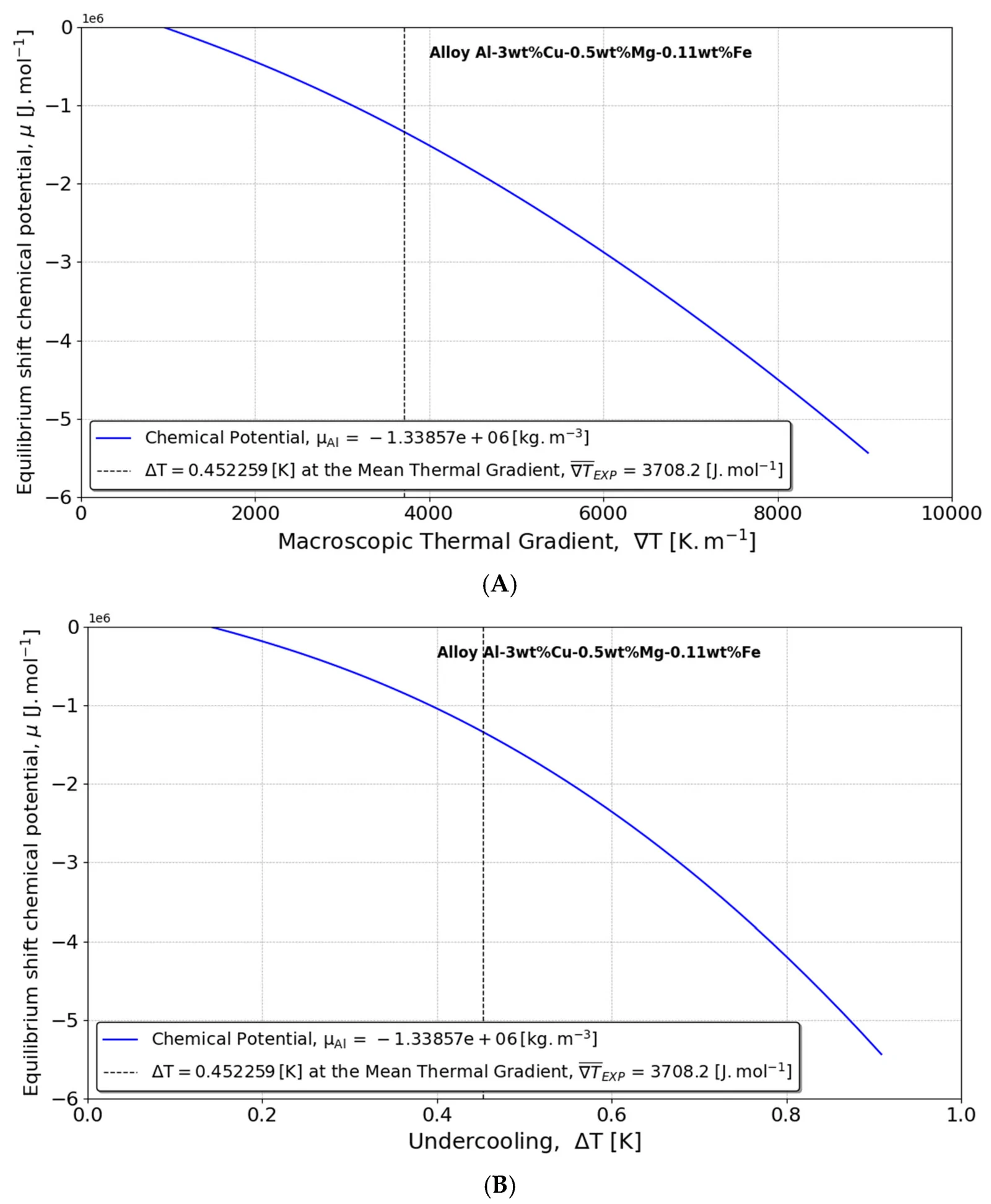
Predicted equilibrium-shift chemical potential against (**A**) the thermal gradient and **(B**) the undercooling.

**Figure 12:**
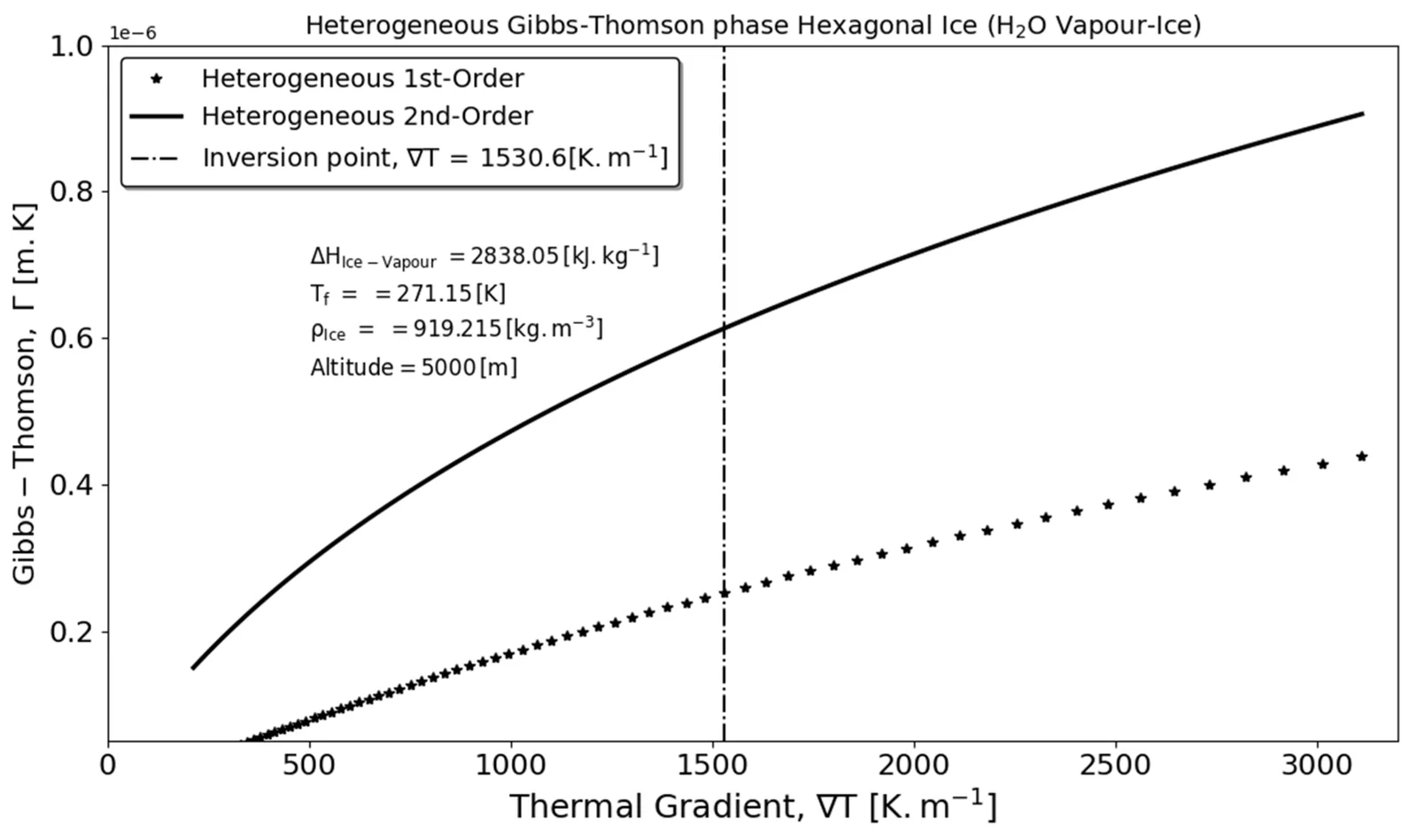
Thermal field tensor (Gibbs–Thomson coefficient) as a function of the thermal gradient for the water vapor–hexagonal ice transformation.

**Figure 13:**
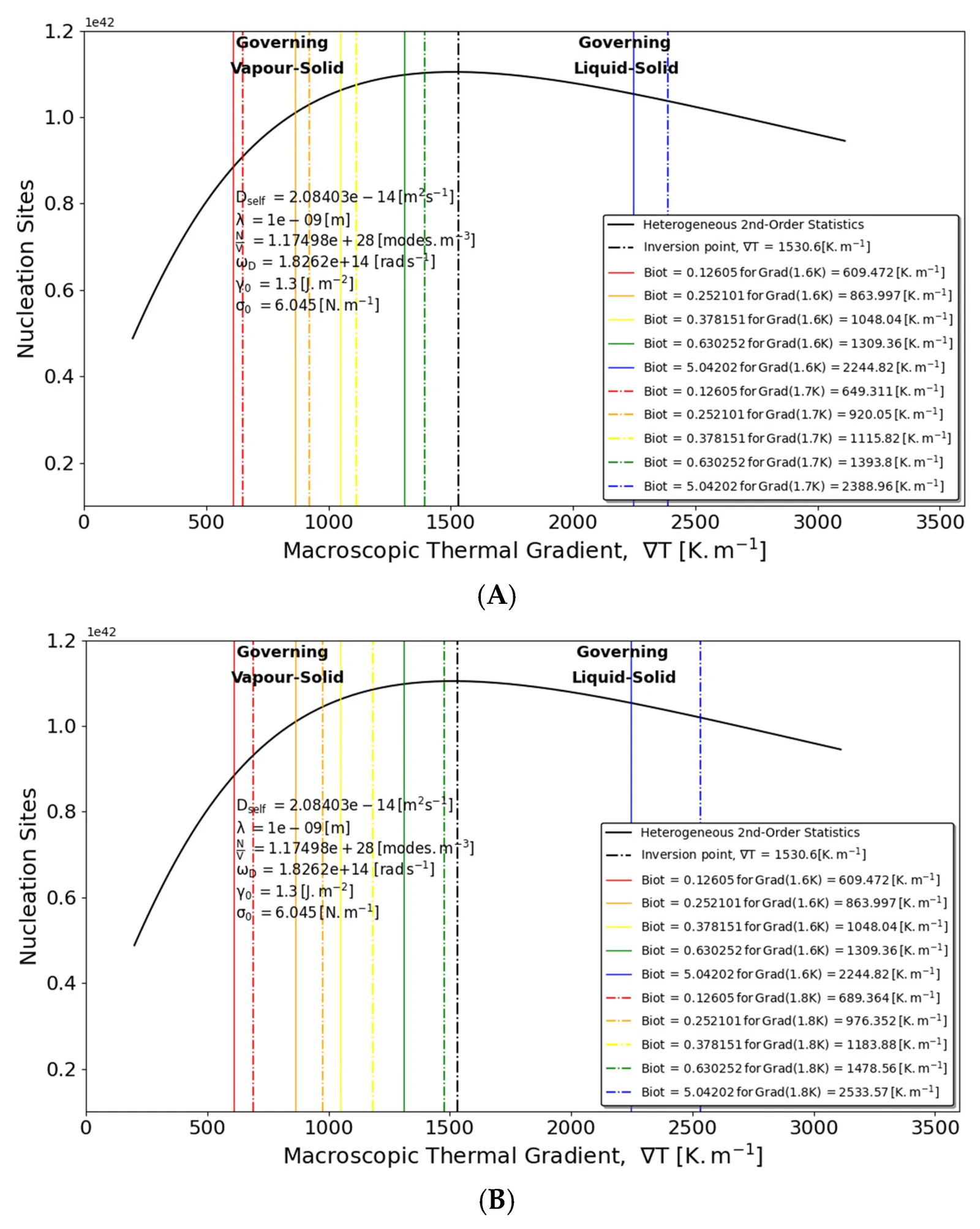

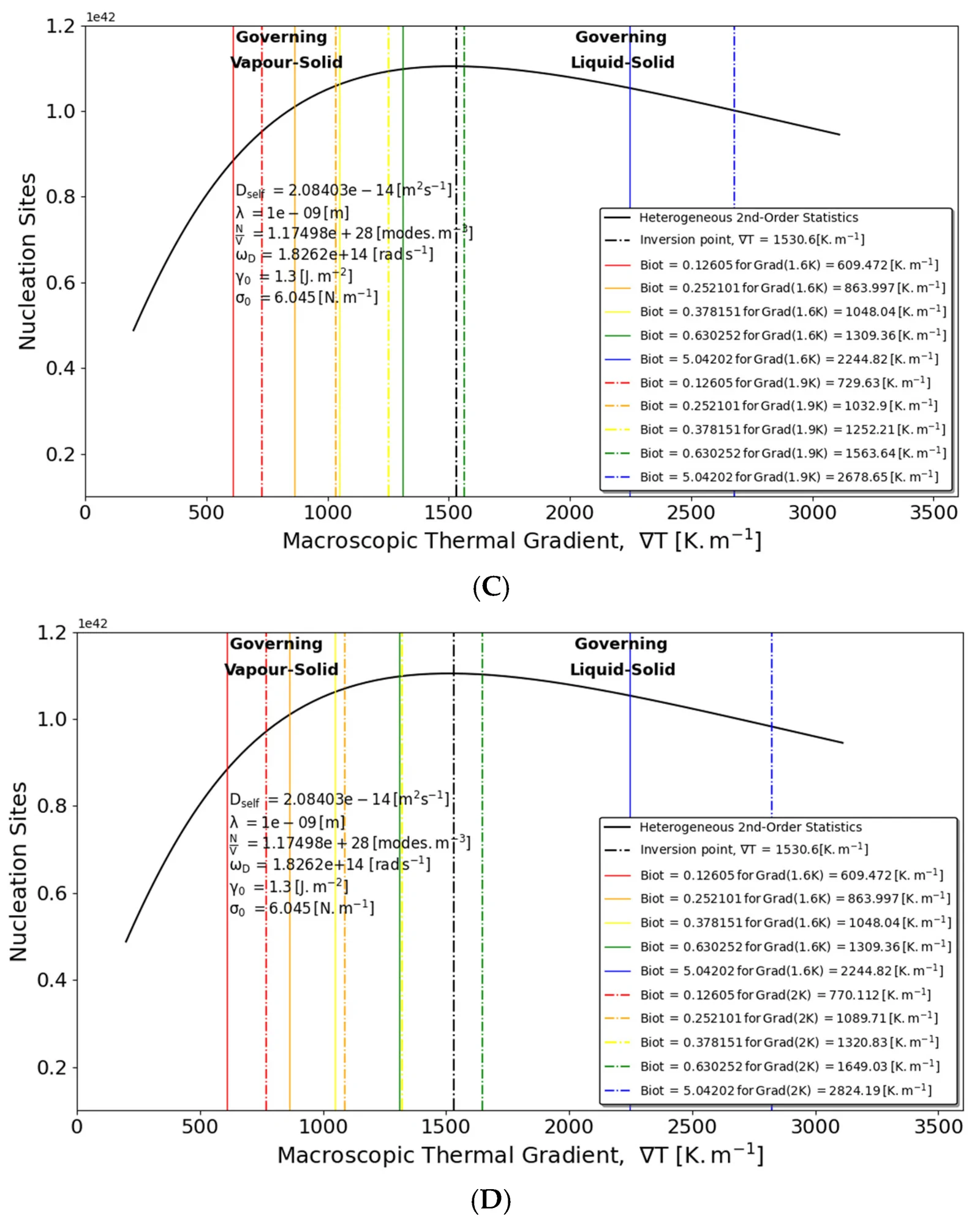
Nucleation rate as a function of the thermal gradient for the formation of hexagonal ice in moist air over a given range of Biot numbers, for superheat values of (**A**) 1.7 K, (**B**) 1.8 K, (**C**) 1.9 K, and (**D**) 2.0 K.

**Table 1:** Calculated nucleation variables as a function of the thermal gradient for the FCC_A1 phase. Alloy composition: Al-3 wt%Cu-0.5 wt%Mg-0.02 wt%Fe.

∇T $K\cdot m^{-1}$	ΔT K	$r_{C.Hom}^{1st}$ m	$r_{C.Het}^{1st}$ m	$\theta^{1st}$ rad	$\frac{\partial f\left(\theta^{1st}\right)}{\partial r}$ 1/m	$r_{C.Hom}^{2nd}$ m	$r_{C.Het}^{2nd}$ m	$\theta^{2nd}$ rad	$\frac{\partial f\left(\theta^{2nd}\right)}{\partial r}$ 1/m	$\sigma_{SL}$ $N\cdot m^{-1}$	Σ $N\cdot m^{-1}$	$\gamma_{SL}$ $J\cdot m^{-2}$
443.579	0.0698834	6.26850 × 10^−6^	7.33440 × 10^−7^	0.306408	−0.02563	6.26850 × 10^−6^	6.26849 × 10^−6^	3.06676	−15.9999	−1.22112	−0.0121204	0.182823
1030.02	0.151674	5.85900 × 10^−6^	1.22400 × 10^−6^	0.47865	−0.14588	5.85900 × 10^−6^	5.85898 × 10^−6^	3.05653	−15.9998	−1.38448	−0.175477	0.370875
2004.31	0.271338	5.38650 × 10^−6^	1.60261 × 10^−6^	0.633015	−0.42139	5.38650 × 10^−6^	5.38648 × 10^−6^	3.0506	−15.9998	−1.58779	−0.378791	0.609973
3016.2	0.382059	5.04000 × 10^−6^	1.77900 × 10^−6^	0.727349	−0.70365	5.04000 × 10^−6^	5.03998 × 10^−6^	3.04819	−15.9998	−1.74856	−0.539564	0.803627
**3708.2**	**0.452259**	**4.85277 × 10^−6^**	**1.84446 × 10^−6^**	**0.773305**	**−0.87855**	**4.85277 × 10^−6^**	**4.85274 × 10^−6^**	**3.04734**	**−15.9998**	**−1.84011**	**−0.631105**	**0.915939**
4112.97	0.49168	4.75650 × 10^−6^	1.87081 × 10^−6^	0.795747	−0.97352	4.75650 × 10^−6^	4.75648 × 10^−6^	3.047	−15.9998	−1.88855	−0.679553	0.976031
5022.88	0.576596	4.56750 × 10^−6^	1.90906 × 10^−6^	0.837649	−1.16827	4.56750 × 10^−6^	4.56748 × 10^−6^	3.04652	−15.9998	−1.98659	−0.777594	1.09912
6115.05	0.672923	4.37850 × 10^−6^	1.93075 × 10^−6^	0.876909	−1.37190	4.37850 × 10^−6^	4.37848 × 10^−6^	3.04625	−15.9998	−2.08878	−0.879779	1.22966
7196.24	0.763415	4.22100 × 10^−6^	1.93715 × 10^−6^	0.90775	−1.54655	4.22100 × 10^−6^	4.22098 × 10^−6^	3.04617	−15.9998	−2.17736	−0.968361	1.34484
8196.26	0.843547	4.09500 × 10^−6^	1.93514 × 10^−6^	0.93126	−1.68847	4.09500 × 10^−6^	4.09498 × 10^−6^	3.0462	−15.9998	−2.25064	−1.04164	1.44164
9038.27	0.90874	4.00050 × 10^−6^	1.92968 × 10^−6^	0.948238	−1.79571	4.00050 × 10^−6^	4.00048 × 10^−6^	3.04626	−15.9998	−2.30709	−1.09809	1.51722

**Table 2:** Calculated nucleation variables as a function of the thermal gradient for the FCC_A1 phase. Alloy composition: Al-3 wt%Cu-0.5 wt%Mg-0.02 wt%Fe.

∇T $K\cdot m^{-1}$	ΔT K	$\frac{{\partial \gamma }_{SL}}{\partial r}$ $J\cdot m^{-3}$	$\Delta S_{V}$ $J\cdot m^{-3}K^{-1}$	$\Gamma^{1st}$ m·K	$\Gamma^{2nd}$ m·K	$\Delta S_{V}$ $J\cdot m^{-3}K^{-1}$	$\frac{\partial \Delta S_{V}}{\partial r}$ $J\cdot m^{-4}K^{-1}$	$\Delta S_{S}$ $J\cdot m^{-3}K^{-1}$	$\Delta S_{C}$ $J\cdot m^{-3}K^{-1}$	$\Delta G_{V}$ $J\cdot m^{-3}$	$\Delta G_{S}$ $J\cdot m^{-2}$	$\Delta G_{C}$ $J\cdot m^{-3}$
443.579	0.0698834	−440,205	−1,023,510	2.56276 × 10^−8^	2.19033 × 10^−7^	−1,023,510	5.99434 × 10^12^	−6,299,140	−10.4645	−71,526.2	0.182823	−0.73129
1030.02	0.151674	−479,403	−2,104,620	9.28244 × 10^−8^	4.4433 × 10^−7^	−2,104,620	1.24147 × 10^12^	−3,160,740	−9.78086	−319,216	0.370875	−1.4835
2004.31	0.271338	−534,742	−2,418,780	2.17423 × 10^−7^	7.30784 × 10^−7^	−2,418,780	3.59340 × 10^11^	−1,970,760	−8.99208	−656,308	0.609973	−2.43989
3016.2	0.382059	−584,554	−2,501,210	3.39841 × 10^−7^	9.62793 × 10^−7^	−2,501,210	1.61315 × 10^11^	−1,530,010	−8.41364	−955,609	0.803627	−3.21451
**3708.2**	**0.452259**	**−615,670**	**−2,524,800**	**4.17091 × 10^−7^**	**1.09735 × 10^−6^**	**−2,524,800**	**1.04046 × 10^11^**	**−1,361,360**	**−8.10108**	**−1,141,860**	**0.915939**	**−3.66379**
4112.97	0.49168	−633,025	−2,533,390	4.5992 × 10^−7^	1.16934 × 10^−6^	−2,533,390	8.21916 × 10^10^	−1,287,470	−7.94037	−1,245,620	0.976031	−3.90412
5022.88	0.576596	−670,186	−2,545,160	5.50379 × 10^−7^	1.31681 × 10^−6^	−2,545,160	4.97170 × 10^10^	−1,162,310	−7.62486	−1,467,530	1.09912	−4.39646
6115.05	0.672923	−712,067	−2,551,940	6.49625 × 10^−7^	1.4732 × 10^−6^	−2,551,940	2.70773 × 10^10^	−1,058,170	−7.30935	−1,717,260	1.22966	−4.91863
7196.24	0.763415	−751,240	−2,554,870	7.39428 × 10^−7^	1.6112 × 10^−6^	−2,554,870	1.34190 × 10^10^	−984,051	−7.04642	−1,950,420	1.34484	−5.37934
8196.26	0.843547	−785,844	−2,555,870	8.16194 × 10^−7^	1.72717 × 10^−6^	−2,555,870	5.00422 × 10^9^	−931,595	−6.83608	−2,156,000	1.44164	−5.76655
9038.27	0.90874	−813,967	−2,556,020	8.76795 × 10^−7^	1.81772 × 10^−6^	−2,556,020	−1.45567 × 10^8^	−895,710	−6.67833	−2,322,750	1.51722	−6.06887

**Table 3:** Calculated nucleation variables as a function of the thermal gradient for the water vapor–hexagonal ice equilibrium-shift transformation.

∇T $K\cdot m^{-1}$	ΔT K	$r_{C.Hom}^{1st}$ m	$r_{C.Het}^{1st}$ m	$\theta^{1st}$ rad	$\frac{\partial f\left(\theta^{1st}\right)}{\partial r}$ 1/m	$r_{C.Hom}^{2nd}$ m	$r_{C.Het}^{2nd}$ m	$\theta^{2nd}$ rad	$\frac{\partial f\left(\theta^{2nd}\right)}{\partial r}$ 1/m	$\sigma_{SL}$ $N\cdot m^{-1}$	Σ $N\cdot m^{-1}$	$\gamma_{SL}$ $J\cdot m^{-2}$
199.74	0.037862	7.54210 × 10^−6^	1.18653 × 10^−6^	0.384316	−0.06230	7.54210 × 10^−6^	7.54208 × 10^−6^	3.05719	−15.9998	−6.10560	−0.06060	1.37370
414.21	0.072991	7.01150 × 10^−6^	1.72165 × 10^−6^	0.543308	−0.23688	7.01150 × 10^−6^	7.01147 × 10^−6^	3.04895	−15.9998	−6.98759	−0.94259	2.46195
511.62	0.087720	6.82200 × 10^−6^	1.86528 × 10^−6^	0.591642	−0.32705	6.82200 × 10^−6^	6.82197 × 10^−6^	3.04707	−15.9998	−7.31885	−1.27385	2.87879
723.16	0.117790	6.48090 × 10^−6^	2.07034 × 10^−6^	0.670422	−0.52160	6.48090 × 10^−6^	6.48086 × 10^−6^	3.04457	−15.9997	−7.93901	−1.89401	3.67234
805.95	0.128972	6.36720 × 10^−6^	2.12479 × 10^−6^	0.694658	−0.59460	6.36720 × 10^−6^	6.36716 × 10^−6^	3.04394	−15.9997	−8.15301	−2.10801	3.95041
1028.23	0.157686	6.10190 × 10^−6^	2.22745 × 10^−6^	0.747801	−0.77830	6.10190 × 10^−6^	6.10186 × 10^−6^	3.04278	−15.9997	−8.66758	−2.62258	4.62867
1100.25	0.166635	6.02610 × 10^−6^	2.25088 × 10^−6^	0.762175	−0.83381	6.02610 × 10^−6^	6.02606 × 10^−6^	3.04252	−15.9997	−8.81871	−2.77371	4.83059
1481.59	0.211689	5.68500 × 10^−6^	2.32669 × 10^−6^	0.822846	−1.09684	5.68500 × 10^−6^	5.68496 × 10^−6^	3.04169	−15.9997	−9.52321	−3.47821	5.78932
1530.60 *	**0.217233**	**5.64710 × 10^−6^**	**2.33229 × 10^−6^**	**0.829206**	**−1.12718**	**5.64710 × 10^−6^**	**5.64706 × 10^−6^**	**3.04163**	**−15.9997**	**−9.60409**	**−3.55909**	**5.90132**
1581.10	0.222895	5.60920 × 10^−6^	2.33736 × 10^−6^	0.835494	−1.15769	5.60920 × 10^−6^	5.60917 × 10^−6^	3.04158	−15.9997	−9.68550	−3.64050	6.01450
1858.17	0.253105	5.41970 × 10^−6^	2.35493 × 10^−6^	0.865877	−1.31259	5.41970 × 10^−6^	5.41967 × 10^−6^	3.04139	−15.9997	−10.10100	−4.05603	6.59894
1918.93	0.259553	5.38180 × 10^−6^	2.35694 × 10^−6^	0.871749	−1.34395	5.38180 × 10^−6^	5.38177 × 10^−6^	3.04137	−15.9997	−10.18590	−4.14088	6.71973
2253.32	0.294051	5.19230 × 10^−6^	2.35986 × 10^−6^	0.900114	−1.50209	5.19230 × 10^−6^	5.19227 × 10^−6^	3.04134	−15.9997	−10.61930	−4.57428	7.34480
2402.88	0.308991	5.11650 × 10^−6^	2.35781 × 10^−6^	0.911008	−1.56577	5.11650 × 10^−6^	5.11647 × 10^−6^	3.04136	−15.9997	−10.79710	−4.75208	7.60531
2822.97	0.349565	4.92700 × 10^−6^	2.34508 × 10^−6^	0.937149	−1.72521	4.92700 × 10^−6^	4.92697 × 10^−6^	3.04150	−15.9997	−11.25340	−5.20839	8.28531
3214.08	0.385750	4.77540 × 10^−6^	2.32743 × 10^−6^	0.956958	−1.85234	4.77540 × 10^−6^	4.77537 × 10^−6^	3.04168	−15.9997	−11.63120	−5.58625	8.86163

* Climate inversion point thermal gradient.

**Table 4:** Calculated nucleation variables as a function of the thermal gradient for the water vapor–hexagonal ice equilibrium-shift transformation.

∇T $K\cdot m^{-1}$	ΔT K	$\frac{{\partial \gamma }_{SL}}{\partial r}$ $J\cdot m^{-3}$	$\Delta S_{V}$ $J\cdot m^{-3}K^{-1}$	$\Gamma^{1st}$ m·K	$\Gamma^{2nd}$ m·K	$\frac{\partial \Delta S_{V}}{\partial r}$ $J\cdot m^{-4}K^{-1}$	$\Delta S_{S}$ $J\cdot m^{-2}K^{-1}$	$\Delta S_{C}$ $J\cdot m^{-3}K^{-1}$	$\Delta G_{V}$ $J\cdot m^{-3}$	$\Delta G_{S}$ $J\cdot m^{-2}$	$\Delta G_{C}$ $J\cdot m^{-3}$
199.74	0.037862	−1,951,220	−12,282,000	2.24620 × 10^−8^	1.42779 × 10^−7^	7.02055 × 10^13^	−51,535,200	−145.128	−465,018	1.37370	−5.49481
414.21	0.072991	−2,157,710	−30,960,300	6.28323 × 10^−8^	2.55889 × 10^−7^	1.83287 × 10^13^	−29,561,400	−134.918	−2,259,820	2.46195	−9.84778
511.62	0.087720	−2,242,740	−33,721,700	8.18109 × 10^−8^	2.99216 × 10^−7^	1.24308 × 10^13^	−25,566,900	−131.271	−2,958,080	2.87879	−11.51520
723.16	0.117790	−2,414,290	−36,721,500	1.21932 × 10^−7^	3.81696 × 10^−7^	6.51308 × 10^12^	−20,496,500	−124.708	−4,325,430	3.67234	−14.68940
805.95	0.128972	−2,477,530	−37,367,200	1.37019 × 10^−7^	4.10598 × 10^−7^	5.29128 × 10^12^	−19,209,800	−122.52	−4,819,330	3.95041	−15.80170
1028.23	0.157686	−2,638,900	−38,442,500	1.75617 × 10^−7^	4.81095 × 10^−7^	3.26130 × 10^12^	−16,735,200	−117.415	−6,061,850	4.62867	−18.51470
1100.25	0.166635	−2,688,950	−38,665,200	1.87536 × 10^−7^	5.02082 × 10^−7^	2.83385 × 10^12^	−16,136,800	−115.956	−6,442,970	4.83059	−19.32240
1481.59	0.211689	−2,939,730	−39,348,300	2.46267 × 10^−7^	6.01731 × 10^−7^	1.44922 × 10^12^	−13,887,000	−109.393	−8,329,610	5.78932	−23.15730
1530.60 *	**0.217233**	**−2,970,480**	**−39,398,900**	**2.53324 × 10^−7^**	**6.13372 × 10^−7^**	**1.33656 × 10^12^**	**−13,674,200**	**−108.663**	**−8,558,740**	**5.90132**	**−23.60530**
1581.10	0.222895	−3,001,870	−39,445,500	2.60492 × 10^−7^	6.25136 × 10^−7^	1.23029 × 10^12^	−13,467,700	−107.934	−8,792,220	6.01450	−24.05800
1858.17	0.253105	−3,169,170	−39,625,700	2.98021 × 10^−7^	6.85881 × 10^−7^	7.81822 × 10^11^	−12,521,200	−104.288	−10,029,500	6.59894	−26.39580
1918.93	0.259553	−3,204,850	−39,652,500	3.05874 × 10^−7^	6.98436 × 10^−7^	7.06487 × 10^11^	−12,347,600	−103.558	−10,291,900	6.71973	−26.87890
2253.32	0.294051	−3,395,640	−39,748,800	3.46957 × 10^−7^	7.63405 × 10^−7^	3.87477 × 10^11^	−11,547,800	−99.912	−11,688,200	7.34480	−29.37920
2402.88	0.308991	−3,478,270	−39,772,100	3.64269 × 10^−7^	7.90481 × 10^−7^	2.82967 × 10^11^	−11,256,900	−98.4535	−12,289,200	7.60531	−30.42120
2822.97	0.349565	−3,702,860	−39,800,400	4.09877 × 10^−7^	8.61159 × 10^−7^	6.78629 × 10^10^	−10,592,800	−94.8071	−13,912,800	8.28531	−33.14120
3214.08	0.385750	−3,903,530	−39,797,700	4.48902 × 10^−7^	9.21061 × 10^−7^	−6.51598 × 10^10^	−10,119,300	−91.8899	−15,352,000	8.86163	−35.44650

* Climate inversion point thermal gradient.

## Data Availability

The data supporting the findings of this study are available from the corresponding author upon reasonable request.
